# The Functions of Grainy Head-Like Proteins in Animals and Fungi and the Evolution of Apical Extracellular Barriers

**DOI:** 10.1371/journal.pone.0036254

**Published:** 2012-05-09

**Authors:** Adam Paré, Myungjin Kim, Michelle T. Juarez, Stuart Brody, William McGinnis

**Affiliations:** 1 Section of Cell & Developmental Biology, University of California San Diego, La Jolla, California, United States of America; 2 Section of Molecular Biology, University of California San Diego, La Jolla, California, United States of America; University of California Riverside, United States of America

## Abstract

The Grainy head (GRH) family of transcription factors are crucial for the development and repair of epidermal barriers in all animals in which they have been studied. This is a high-level functional conservation, as the known structural and enzymatic genes regulated by GRH proteins differ between species depending on the type of epidermal barrier being formed. Interestingly, members of the CP2 superfamily of transcription factors, which encompasses the GRH and LSF families in animals, are also found in fungi – organisms that lack epidermal tissues. To shed light on CP2 protein function in fungi, we characterized a *Neurospora crassa* mutant lacking the CP2 member we refer to as *grainy head-like* (*grhl*). We show that *Neurospora* GRHL has a DNA-binding specificity similar to that of animal GRH proteins and dissimilar to that of animal LSF proteins. *Neurospora grhl* mutants are defective in conidial-spore dispersal due to an inability to remodel the cell wall, and we show that *grhl* mutants and the long-known *conidial separation-2* (*csp-2*) mutants are allelic. We then characterized the transcriptomes of both *Neurospora grhl* mutants and *Drosophila grh* mutant embryos to look for similarities in the affected genes. *Neurospora grhl* appears to play a role in the development and remodeling of the cell wall, as well as in the activation of genes involved in defense and virulence. *Drosophila* GRH is required to activate the expression of many genes involved in cuticular/epidermal-barrier formation. We also present evidence that GRH plays a role in adult antimicrobial defense. These results, along with previous studies of animal GRH proteins, suggest the fascinating possibility that the apical extracellular barriers of some animals and fungi might share an evolutionary connection, and that the formation of physical barriers in the last common ancestor was under the control of a transcriptional code that included GRH-like proteins.

## Introduction

Grainy head (GRH) transcription factors are crucial for many aspects of development. For instance, *Drosophila* GRH (also called Elf-1 or NTF-1) regulates development of the epidermis and head skeleton [1], [2], wound healing [3]–[6], neuroblast proliferation [7], [8], early embryonic patterning [9], [10], and tracheal-tube morphology [11]. However, the functions of GRH family proteins with respect to epidermal-barrier formation and wound healing have received the most attention, as these functions appear to be widely conserved in animals.

*Drosophila grh* mutant embryos have slack and fragile cuticles, as well as “grainy” and discontinuous head skeletons [1], [2], [12]. Null mutations are lethal, as the embryos fail to develop past the embryonic/larval transition point due to their extremely fragile epidermal barriers. These phenotypes clearly point to defects in the formation of chitin-based cuticular structures in *grh* mutant embryos. These defects are likely due to lowered epidermal expression of a wide variety of genes, among them *Ddc*, which encodes dopa decarboxylase, an enzyme required to generate the reactive quinone molecules used to cross-link chitin fibers and proteins in the *Drosophila* cuticle [1], [3]. Furthermore, *grh* embryos are permeable to exogenously applied dyes [6], and the removal of GRH from imaginal disc cells results in reduced expression of at least two cell-adhesion genes [13]. These findings suggest that the paracellular integrity of the epithelial barrier underlying the cuticle becomes compromised in *Drosophila grh* mutants. In addition to the developmental functions of GRH in *Drosophila*, it is also necessary for the proper expression of several cuticular-barrier genes that are activated during the regenerative process following epidermal wounding [3]–[5].

GRH family proteins are also important for epidermal-barrier formation in the distantly related invertebrate *C. elegans*. RNAi targeted against *Ce-Grh-1* results in embryos with a fragile and puckered hypodermis – a similar phenotype to that seen in *Drosophila* [14]. Ce-Grh-1 binds the same palindromic consensus DNA sequences as *Drosophila* GRH, and the *Ddc* gene in *C. elegans* has GRH binding sites upstream of its promoter [14]. Strikingly, conservation of GRH family transcription factor function extends to vertebrates as well, despite vast differences in the structural components of epidermal barriers between and within protostome and deuterostome animals. In *Xenopus laevis*, expression of a dominant negative form of XGRHL1 leads to a malformed epidermis, partly due to lowered expression of keratin [15]. The mouse genome contains three paralogs of GRH, encoded by the genes *Grainy head-like 1, −2, and −3* (*Grhl1, −2,* and *−3*), which are all expressed in the surface ectoderm during development [16]–[18]. Mutations of both mouse *Grhl1* and *Grhl3* genes result in a malformed epidermis. *Grhl3* (also known as *Get1*) knockout mutants display the most severe phenotypes, including abnormal epithelial morphology in both the epidermis and bladder, impaired wound healing, defective extracellular lipid processing, increased permeability to exogenous dyes, and severe postnatal water loss, as well as defects in neural-tube and eyelid closure [16], [19]–[24]. *Grhl1*-deficient mice display delayed coat growth, thickened paw skin, and hair loss due to poor anchoring of hair shafts within follicles [25]. *Grhl2* appears to regulate neural-tube closure as well as E-cadherin expression [26]. Furthermore, all three mouse Grhl transcription factors have been shown to bind preferentially to the same consensus DNA sequences as *Drosophila* and *C. elegans* GRH proteins [27].

While the DNA-binding specificity of GRH family proteins has been conserved between protostome and deuterostome animals, the downstream effectors of GRH-like proteins in distantly related species do not appear to be homologous, but instead carry out analogous functions suited to the specific barrier being generated or regenerated after wounding. For instance, the epidermal defects in *Grhl3*-deficient mice correlate with reduced levels of *transglutaminase 1* transcription (which has upstream GRH binding sites), as well as reduced transcription for many genes that are structural barrier components of differentiated corneocytes [19], [21]. Transglutaminase 1 is an enzyme necessary for the cross-linking of keratin and other proteins in the mammalian epidermis, and it plays an analogous role to that of dopa decarboxylase in the *Drosophila* cuticle. In sum, there exists a high-level functional conservation of GRH proteins as regulators of epidermal integrity and wound healing in both protostome and deuterostome animals (which diverged approximately 700 million years ago), despite the significant structural differences in barrier composition across the animal kingdom. This functional conservation is reminiscent of other cases in which high-level transcription factor function has been conserved over great evolutionary time (e.g., Hox genes, *Pax6*/*eyeless,* and *Nkx2.5/tinman* in body-axis, eye, and heart specification, respectively) despite the drift of specific downstream effectors.

Since the function of GRH-like proteins in epidermal-barrier formation and wound healing appears well conserved in triploblastic animals, we were interested in determining what role GRH-like proteins might be playing in more distantly related organisms. GRH family proteins (along with the related LSF family proteins) belong to the CP2 superfamily of transcription factors, members of which are only found in the opisthokont lineage, which includes Metazoa (Animals), Fungi, and several closely related sister-species [28]. Considering the fact that Fungi utilize a very different type of extracellular physical barrier (the cell wall) compared with animals, we thought that by studying the role of CP2 superfamily transcription factors in Fungi we might shed some light on the origins of transcriptional control of physical-barrier formation in the opisthokont ancestor. Towards this end, we have characterized the function of the CP2 superfamily gene in the ascomycete fungus *Neurospora crassa* using microarray and phenotypic analyses. We show that the loss of this *Neurospora* gene, which we call *grainy head-like* (*grhl*), leads to a developmental defect in cell wall remodeling during conidial development, which is associated with the down-regulation of numerous genes predicted to encode abundant components of the cell wall. We also carried out microarray and phenotypic analyses of *Drosophila grh* mutants, and we present evidence that, in addition to its crucial role in cuticular- and epidermal-barrier formation, GRH may also be involved in microbial defense during adulthood in *Drosophila*. Our results suggest an ancestral role for CP2 superfamily proteins as regulators of extracellular-barrier formation in opisthokont ancestors.

## Results

### Sequence Analyses Suggest Fungal CP2 Proteins are More Functionally Similar to Animal GRH Proteins than to Animal LSF Proteins

The CP2 superfamily is composed of the GRH and LSF families of transcription factors. A comprehensive review of the functions of LSF-like proteins is beyond the scope of this paper, but there appears to be little overlap between the biological roles of the GRH and LSF families in animals [29], and the two families have diverged greatly in their modes of DNA binding [30], [31]. It is clear that the last common ancestor of Metazoa and Fungi possessed at least one CP2 superfamily protein, although phylogenetic analysis indicates that fungal CP2 superfamily proteins form a separate outgroup with respect to metazoan GRH and LSF family proteins [28]. With few exceptions, all sequenced metazoan genomes possess one or more copies of both GRH and LSF family proteins. Among the Fungi, only ascomycete and zygomycete genomes encode a CP2 superfamily protein (or multiple paralogs), while known basidiomycete genomes do not. Some ascomycetes (e.g., *Saccharomyces cerevisiae*) appear to have lost the CP2 superfamily. The unicellular sister-group organisms *M. brevicollis* (a choanoflagellate) and *C. owczarzaki* (a filasterean) both contain single CP2 superfamily proteins [32], [33].

**Figure 1:**
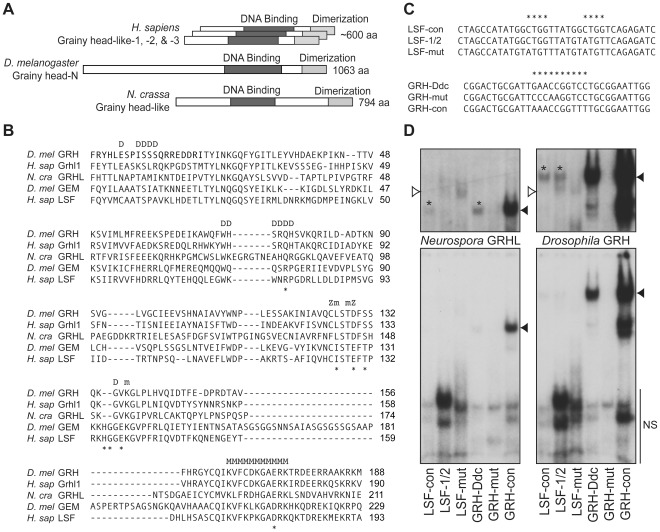
*Neurospora* GRHL has a similar DNA binding specificity as *Drosophila* GRH. (**A**) The *Neurospora* GRHL protein shares sequence similarity with both *Drosophila* GRH and mammalian Grhl proteins, as well as with LSF family proteins [28]. The areas of highest similarity include the region containing the GRH DNA-binding domain, and a region near the C-terminus containing the GRH dimerization domain [30]. (**B**) A comparison of the DNA-binding domain of a representative fungal CP2 superfamily protein (*N. cra* GRHL) with those of the *Drosophila* and human GRH family proteins (*D. mel* GRH and *H. sap* Grhl1) and LSF family proteins (*D. mel* GEM and *H. sap* LSF). Amino acid residues predicted to be important for DNA interactions based on comparisons with p53 transcription factors [34] are marked above the alignment as follows: “D” – dimerization; “Z” – zinc-binding; “m” – minor-groove interaction; and “M” – major-groove interaction. Residues that distinguish GRH family from LSF family proteins are indicated below the alignment with asterisks. The indicated amino acids in nearly all known fungal CP2 superfamily proteins are identical to the *Neurospora* residues. (**C**) Oligonucleotides used in the gel-shifts. Bases that include the LSF or GRH optimal consensus binding sites are indicated with asterisks. (**D**) Gel-shift assays testing *Drosophila* GRH and *Neurospora* GRHL binding to the oligonucleotides in (C). The bottom panels were exposed for 16.5 h, and the top panels were exposed for 75 h. Specific bands are indicated with black arrowheads, and weak specific bands are also highlighted with asterisks in the top panels. Nonspecific (NS) bands were also detected in the no-protein-template negative controls (data not shown), and they are indicated with white arrowheads in the top panels and with a bar in the bottom panels.

Although a recently published phylogenetic analysis using gap-free alignments of near full-length protein sequences showed that fungal CP2 superfamily proteins are roughly equally related to both the GRH and LSF protein families [28], we decided to look more closely at the DNA-binding domain sequences of extant opisthokont CP2 superfamily proteins to identify specific residues that might be characteristic of GRH or LSF proteins. An alignment between the DNA-binding domains of two GRH family proteins (*D. melanogaster* GRH and *H. sapiens* Grhl1), two LSF family proteins (*D. melanogaster* GEM and *H. sapiens* LSF), and a representative fungal CP2 superfamily protein (referred to as *Neurospora* Grainy head-like, or GRHL, for reasons described below) highlights the extensive sequence conservation throughout this domain (Figures 1A and B). It has been predicted that part of the region containing the DNA-binding domain of CP2 superfamily proteins adopts a similar tertiary structure to the DNA-binding domain of p53 [34], which has a well-characterized three-dimensional structure. Strikingly, the identity of eight amino acid residues at and around positions predicted to be crucial for DNA binding, based on mapping to the p53 structure (i.e., major- and minor-groove contacts, zinc-binding residues, and residues involved in dimerization) suggest that the DNA-binding properties of fungal CP2 proteins might be more similar to GRH than to LSF family proteins. For example, relative to positions 194–198 of the *Neurospora* GRHL DNA-binding domain (a region predicted to be involved in major-groove interaction) the same amino acid sequence GAERK is found in nearly all available metazoan GRH and fungal GRHL ortholog sequences, while the sequence GADRK is found in all available metazoan LSF sequences (Figure 1B). Similarly, in three other regions predicted to be important for DNA binding (positions 81–84, 142–147 and 150–153), we find that some *Neurospora* GRHL amino acid residues are identical to those found in nearly all fungal CP2 proteins and metazoan GRH proteins, but they differ from those found in LSF proteins (indicated with asterisks in Figure 1B). Based on these observations, we hypothesized that the DNA-binding characteristics of the *Neurospora* GRHL protein might be more similar to those of animal GRH family proteins than animal LSF family proteins. Another indication that fungal CP2 superfamily proteins might be more functionally similar to animal GRH proteins is that fungal CP2 superfamily proteins all lack SAM oligomerization domains – animal GRH proteins also lack SAM domains, but all known animal LSF family proteins possess SAM domains [28].

### The *Neurospora* CP2 Protein GRHL and *Drosophila* GRH have Similar DNA-binding Specificities

To test whether a fungal CP2 proteins binds DNA similarly to animal GRH family proteins, we decided to study the *Neurospora crassa* CP2 superfamily protein. We chose *Neurospora* as our model organism because it is a fairly typical representative of a filamentous ascomycete fungus, there exist a number of molecular tools to work with (including gene-knockout technologies), and it has a fully sequenced genome. The *Neurospora crassa* genome possesses a single CP2 superfamily gene (designated as NCU06095), which has been called *grainy-head homolog* (*ghh*) [35], but which we will hereafter refer to as *grainy head-like* (*grhl*) or *csp-2*, for the reasons described in the preceding and following sections. Using RT-PCR and primers specific to the predicted start and stop sites, we cloned and sequenced the full-length *grhl* coding region and found the sequence and exon structure to be identical to that in the Broad Institute *Neurospora* database. No splice variants were detected, although we cannot rule out the possibility of *grhl* transcripts that include additional upstream exons or alternate 3’UTRs.

We synthesized full-length *Neurospora* GRHL protein in order to characterize its DNA-binding properties using gel-shift analyses. It has been shown that *Drosophila* and *C. elegans* GRH family proteins can both bind with high affinity as homodimers to the palindromic DNA sequence ACCGGTT from the *Ddc* promoter [14], [30] and that the optimal consensus binding site for murine GRH family proteins contains the palindromic DNA sequence AACCGGTT [19], [27]. Mammalian LSF has been shown to bind as a tetramer to DNA fragments containing the sequence CTGG-N_6_-CTGG; LSF does not bind to DNA fragments containing a GRH site from the *Ubx* promoter [31]. On the other hand, *Drosophila* GRH can weakly bind to both full-length LSF sites and CTGG half-sites [31]. Therefore, we tested the ability of the *Neurospora* GRHL protein to bind DNA oligonucleotides containing one of the following sites: the endogenous GRH binding site (GRH-Ddc), a mutated GRH binding site (GRH-mut), or the consensus GRH binding site (GRH-con) (Figure 1C). We also tested the ability of the GRHL protein to bind DNA oligonucleotides containing one of the following sites: the endogenous LSF consensus site (LSF-con), an LSF half-site (LSF-1/2), or a mutated LSF site (LSF-mut) (Figure 1C). The binding of full-length *Drosophila* GRH protein to these oligonucleotides was tested as a comparison.

*Drosophila* GRH bound DNA sequences as previously reported [30], [31], interacting strongly with the GRH-Ddc and GRH-con oligonucleotides, but not with the GRH-mut oligonucleotide (Figure 1D, right panels). *Drosophila* GRH also bound very weakly to both the LSF-con and LSF-1/2 oligonucleotides, but not to the mutated LSF-mut oligonucleotide (Figure 1D, top right panel). *Neurospora* GRHL bound with a similar specificity as *Drosophila* GRH, albeit with apparent lower affinity. GRHL bound strongly to the GRH-con oligonucleotide, weakly to the GRH-Ddc oligonucleotide, and very weakly to the LSF-con oligonucleotide (Figure 1D, left panels). Considering these results, along with the similarities in their DNA-binding domain sequences, we conclude that the last common ancestor of opisthokonts possessed a CP2 superfamily protein with a similar DNA-binding specificity to existing metazoan GRH family proteins.

### Phenotypes of the *Neurospora grhl* Knockout Mutants

The fungus *Neurospora crassa* has a simple cellular organization and life cycle compared with most animals and plants (for an in-depth treatment on the subject, see [36]). The most visually obvious phase of the *Neurospora* life cycle is asexual proliferation – single spores (conidia) germinate on a food source and form a densely interwoven mat of thread-like mycelia, which spreads quickly to form a colony. *Neurospora* colonies exist as syncytial collections of “cells” which share a common extracellular barrier – the cell wall. While there are regularly spaced septa along the length of the mycelial and hyphal axes, these divisions are not complete, and the “cells” use vigorous cytoplasmic streaming to move nutrients and other molecules throughout the colony. After about a day (and every day after that, according to a circadian rhythm) aerial hyphae grow up and away from the food source and bud off chains of new conidia. These conidial chains become quite delicate as they mature, as the thick cross-walls between individual conidia are remodeled into thin, easily broken connectives – this allows mature conidia to readily detach and disperse to found new colonies.

**Figure 2:**
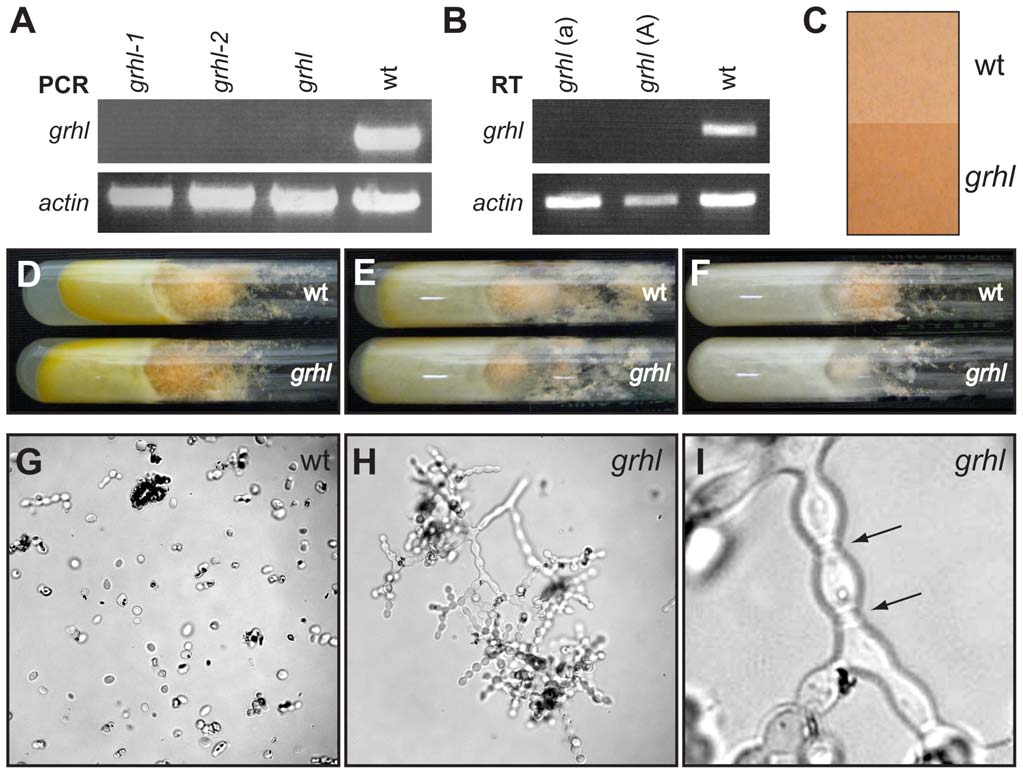
*Neurospora grhl* mutants display a conidial-separation phenotype. (**A**) PCR verification that the *grhl* locus is deleted in two independently generated *grhl* knockout strains (*grhl-1* and *grhl-2*) and in a knockout obtained from the FGSC (*grhl*). The *actin* locus was amplified as a control. (**B**) RT-PCR demonstrates that *grhl* transcripts are not being produced in the FGSC *grhl* knockout strains (mating types a or A). Similar results were obtained using total RNA from conidia or mycelia. Transcripts from the *actin* gene were amplified as a control. (**C**) Mutant *grhl* strains reach full pigmentation more quickly than wild type. Colonies were grown on Petri dishes for 48 h at room temperature in a 12 h light/dark cycle. Approximately 4 cm^2^ of each mature conidiating colony are shown here. (**D–F**) *Neurospora grhl* and wild-type strains have similar growth rates and eventually reach equal pigmentation levels. Shown are Vogel’s agar slants with colony growth after 5 days at 30°C in constant light (D), a 12 h light/dark cycle (E), or constant dark (F). (**G–I**) *Neurospora grhl* strains display a pronounced conidial-separation phenotype. (G) Wild-type conidial chains readily disperse in glycerol to yield individual spores. (H) Mutant *grhl* conidial chains remain intact in glycerol, even after vigorous agitation. (I) A close-up view of a *grhl* conidial chain demonstrating that the conidia remain connected by thick connectives (arrows).

*Neurospora* strains containing precise deletions of the entire *grhl* coding region were obtained from the Fungal Genetics Stock Center (FGSC) for both mating type (mat) backgrounds: FGSC13563 (mat A) and FGSC13564 (mat a). In addition, we created multiple independently derived *grhl* knockout strains using targeted homologous recombination to replace the *grhl* locus with a hygromycin cassette. The phenotypes of these mutant strains were indistinguishable from those of the deletion mutant stocks obtained from the FGSC, indicating that the phenotypes described below are indeed due to the loss of *grhl* function. PCR amplification of a region within the *grhl* locus verified that all strains were indeed lacking the *grhl* gene (Figure 2A). Furthermore, RT-PCR amplification of a region of the *grhl* mRNA yielded no product when RNA from *grhl* mutants was used as template, compared with robust detection of *grhl* transcripts using wild-type RNA as template (Figure 2B). Transcripts from the *grhl* gene were readily detectable by RT-PCR using wild-type RNA templates from either pure mycelial samples or samples of aerial hyphae and conidia (the latter yielding slightly stronger amplifications; data not shown), which suggests the GRHL transcription factor is expressed in most *Neurospora* cell types during asexual proliferation.

Mutant *grhl* strains are viable and can be propagated asexually as homokaryonic colonies (i.e., all nuclei in the colony are clonal) on minimal media. Both *grhl* mating-type strains can serve as males or females in sexual crosses to wild-type or *grhl* strains of the opposite mating type, indicating that *grhl* function is dispensable for sexual reproduction (data not shown). The *grhl* strains appear quite healthy and in many ways are indistinguishable from wild type, at least under laboratory conditions (Figures 2D–F).

The *grhl* mutant strains display a slightly altered circadian rhythm [35], develop orange pigmentation slightly more quickly than wild type (Figure 2C), and sometimes have paler mycelia than wild type (data not shown). However, the most striking phenotype of *grhl* mutants is a pronounced conidial-separation defect. In *grhl* strains, conidial chains fail to completely separate, even upon physical stress or immersion in liquid (Figures 2G–I). This phenotype is identical to that observed in the *conidial separation* mutants *csp-1* and *csp-2*, whose phenotypes have been investigated in some detail. It was shown that the *csp* mutant strains begin conidial development normally; however, the chitinous cross-walls between adjacent conidia do not become remodeled into thin connectives, precluding conidial separation [37]. This phenotype was correlated with a decrease in the autocatalytic activity of the *Neurospora* cell wall, which was hypothesized to be due to the loss of secreted enzymes such as chitinase [38].

While *csp-1* and *csp-2* have long been popular background strains for *Neurospora* researchers (they help prevent the cross-contamination of stocks), the nature of the mutant genes responsible for these phenotypes remained unknown for many years. Recently, it was shown that *csp-1* (NCU02713) encodes a zinc-finger transcription factor on chromosome 1 [39]. However, nature of the gene underlying the *csp-2* phenotype remained unclear, except that it mapped to chromosome 7 between the genes *thi-3* and *ace-8* [40] – precisely the region where *grhl* is located. Therefore, we believed that a lesion in the *grhl* gene might be responsible for the *csp-2* phenotype.

We first carried out genetic complementation tests with the recessive alleles to test whether *csp-2* and *grhl* are allelic. Different *Neurospora* strains can fuse to form syncytial heterokaryonic colonies containing nuclei from both parental strains; these fused colonies are often able to grow in conditions that their parents cannot, as each type of nucleus will complement the requirements of the other. For instance, fused colonies from different nutritional-auxotroph parents can survive on minimal media, which can be taken advantage of to test for genetic complementation at another non-selectable locus. Using standard sexual-crossing procedures, *csp-2* and *grhl* mutations were placed into different auxotrophic backgrounds (*inos* and *his-3*, respectively), and conidia from each strain were combined on minimal media. We found that all viable heterokaryonic fusings resulted in colonies that still displayed the conidial-separation phenotype, demonstrating that *csp-2* and *grhl* mutant alleles fail to complement (see Materials and Methods for details). To assay for the basis of the non-complementation, we sequenced the *grhl* open reading frame of the *csp-2^[FS590]^* allele, and found a one base pair deletion in codon S509. This mutation would be predicted to result in a premature stop codon after 14 out-of-frame codons, leading to the removal of the proper 286 C-terminal amino acids of the GRHL protein (Figure S1). Therefore, we conclude that *grhl* and *csp-2* are allelic and that the conidial-separation phenotype observed in *grhl* strains is due to a reduction in the autocatalytic activity of the cell wall [38], which in turn precludes remodeling of the cross-walls between adjacent conidia [37].

### Microarray Profiling of *Neurospora grhl* Knockout Mutants

To determine the genes directly and indirectly under the control of GRHL in *Neurospora*, we carried out microarray-based transcriptome profiling of three different sample types: 1) MYC – actively growing pure mycelial samples; 2) AHC – aerial hyphae and conidia from 48 h old colonies; and 3) ALL – all cell types from 48 h old colonies. We only describe the results from the AHC samples, as these are the cell types that displayed the conidial-separation phenotype (see the Materials and Methods section for the accession numbers of the MYC and ALL microarray datasets).

Of the 10,526 genes that were probed on the microarray, 167 were seen to be misregulated in the *grhl* AHC samples at a False Discovery Rate (FDR) threshold of less than 0.01 (meaning 1%, or about 2 of these genes, are expected to be false positives). This threshold roughly corresponds to a greater than twofold change in expression up or down relative to wild-type levels. Nearly equal numbers of genes were seen to be up- or down-regulated (84 and 83 genes, respectively), and verification of microarray fold-change directionality for ten genes using quantitative RT-PCR is shown in Figure S2. Up-regulated genes on the *grhl* AHC microarrays are shown in Figure S3, the largest classes of which include genes involved in nitrogen, sulfur, and selenium metabolism, as well as genes involved in membrane transport and cellular import. As we were interested in finding commonalities between the gene products activated by GRH-like transcription factors in animals and fungi, we focused on the down-regulated genes on the *grhl* AHC microarrays.

### Highly Enriched FunCat Categories of the Down-regulated Genes from the *Neurospora grhl* AHC Microarrays

In order to parse microarray results, researchers often use the Gene Ontology (GO) functional annotation system (www.geneontology.org) to look for highly enriched classes of genes. As a comprehensive GO annotation of the *Neurospora crassa* genome did not exist at the time of these analyses, we used an alternative classification system – The Functional Catalogue (FunCat) – for which there did exist a high-quality annotation for Neurospora genes [41]. For the 83 genes that were seen to be significantly down-regulated, there were highly significant enrichments in five FunCat categories (Table 1). Three of these categories are composed of genes involved in amino acid metabolism – specifically that of cysteine, phenylalanine, and tryptophan. A fourth category, “C-compound and carbohydrate transport”, is composed of membrane transport proteins. The fifth highly significant category found was “disease, virulence, and defense”, which is composed of genes predicted to be involved in fungal pathogenicity, defense against other organisms, and certain stress responses.

**Table 1:** Enriched functional categories for the down-regulated *Neurospora grhl* genes and the misregulated *Drosophila grh* genes.

Down-regulated genes from *Neurospora grhl* AHC samples			
Enriched FunCat Categories	FunCat ID	# of genes	p-value
metabolism of the cysteine - aromatic group	01.01.09	6	3.07E−04
metabolism of phenylalanine	01.01.09.04	3	1.63E−03
C-compound and carbohydrate transport	20.01.03	5	1.76E−03
degradation of tryptophan	01.01.09.06.02	2	3.44E−03
disease, virulence, and defense	32.05	6	4.13E−03
**Misregulated genes from ** ***Drosophila grh*** ** samples**			
**Enriched GO Biological Process Categories**	**GO term ID**	**# of genes**	**p-value**
carbohydrate metabolic process	5975	244	1.06E−06
chitin metabolic process	6030	77	2.11E−06
defense response	6952	117	2.67E−06
response to biotic stimulus	9607	109	2.98E−06
aminoglycan metabolic process	6022	95	5.55E−06
response to other organism	51707	104	6.35E−06
immune response	6955	121	1.57E−05
polysaccharide metabolic process	5976	102	2.17E−05
humoral immune response	6959	74	5.40E−05
response to stress	6950	347	8.36E−05
**Enriched GO Molecular Function Categories**	**GO term ID**	**# of genes**	**p-value**
structural constituent of cuticle	42302	96	1.02E−15
structural constituent of chitin-based cuticle	5214	92	2.32E−14
serine-type endopeptidase activity	4252	165	7.63E−14
serine hydrolase activity	17171	185	1.54E−13
serine-type peptidase activity	8236	183	1.85E−13
peptidase activity, acting on L-amino acid peptides	70011	352	8.71E−09
endopeptidase activity	4175	274	1.96E−08
structural constituent of chitin-based larval cuticle	8010	35	4.81E−08
peptidase activity	8233	361	1.17E−07
chitin binding	8061	66	1.85E−07
polysaccharide binding	30247	87	4.75E−07

**(Top)** Enriched Functional Catalogue (FunCat) categories for the 83 significantly down-regulated (FDR <0.01) genes from the *Neurospora grhl* AHC microarrays. **(Middle and Bottom)** The top enriched Gene Ontology (GO) “Biological Process” and “Molecular Function” categories for all misregulated genes from the *Drosophila grh^IM^* embryo microarrays.

We could find no direct connections in the literature between cysteine metabolism and barrier formation in animals. However, most amino-acid-metabolism networks are interlinked, and three of these genes (NCU05499, NCU09183, and NCU01402) are also part of the significantly enriched phenylalanine- and tryptophan-metabolism FunCat categories, for which there are some intriguing connections to barrier formation in animals. Melanization reactions in *Drosophila* are used to harden and cross-link cuticular structures, and are known (at least in the epidermis) to rely on GRH for activation [3]. The reactive quinone molecules used to carry out these processes are derivatives of dopamine, which is itself a derivative of the amino acids tyrosine and phenylalanine (for a review see [42]). It is possible that an ancestral role in phenylalanine regulation by GRH-like transcription factors could have been co-opted by cuticle-forming animals for use in cross-linking apical extracellular barriers. As for the last amino-acid-related FunCat category, “degradation of tryptophan”, there is some evidence that it is a general mechanism of all cells to degrade tryptophan in response to infection, which is used as a means to slow microbial growth through tryptophan deprivation [43]. If true, this function would link tryptophan degradation to the fifth FunCat category – “disease, virulence, and defense” – the presence of which we found especially intriguing, due to the numerous documented connections in animals between physical epidermal barriers and chemical defense against pathogens [44], [45].

### Down-regulated Genes from the *Neurospora grhl* AHC Microarrays

**Figure 3:**
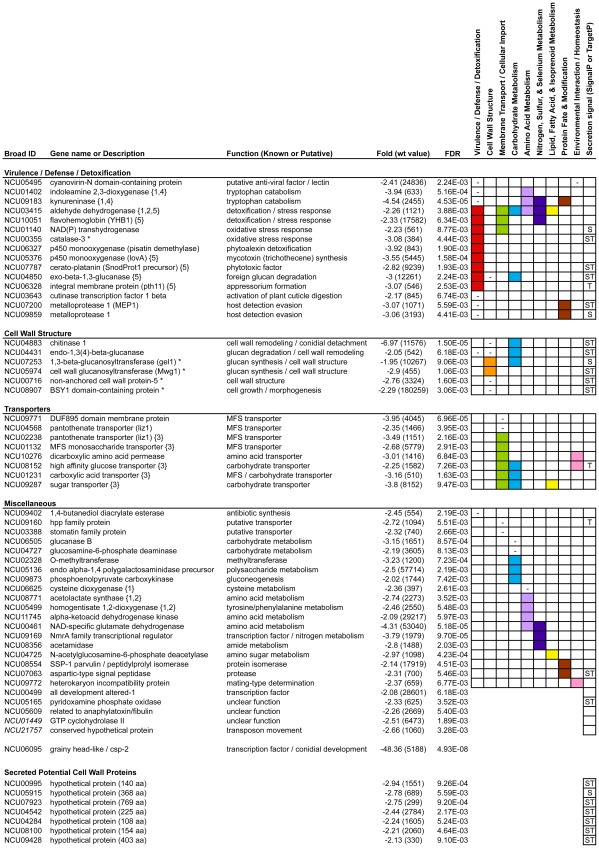
Down-regulated genes from the *Neurospora grhl* Aerial Hyphae and Conidia microarray samples. A manual classification of the significantly down-regulated genes from the *Neurospora grhl* AHC microarrays. **“Broad ID”** entries correspond to the gene IDs found in the Broad Institute *Neurospora crassa* database. The two italicized entries in this column refer to probes that do not correspond to genes in the Broad database, but which correspond to genes in the MIPS database. **“Gene name or Description”** and **“Function”** entries were based on the annotations found in the Broad and MIPS databases, as well literature and homology searches. Numbers in curly brackets indicate genes that belong to one of the five highly enriched FunCat categories: {1} metabolism of the cysteine - aromatic group, {2} metabolism of phenylalanine, {3} C-compound and carbohydrate transport, {4} degradation of tryptophan, and {5} disease, virulence and defense. Entries with asterisks encode experimentally verified components of the *Neurospora* cell wall [46]. **“Fold (wt value)”** entries indicate the fold changes observed in *grhl* mutant aerial hyphae and conidia (relative to wild type); wild-type microarray fluorescence values are shown in parentheses (the background level was ∼100 units). **“FDR”** entries indicate the False Discovery Rate values calculated for each gene; only genes with FDR values less than 0.01 are shown. **Columns 1–9** of the grid represent a simplification of the FunCat classification system; solid-colored blocks indicate those genes are classified in the corresponding FunCat categories; dashes indicate that we found evidence in the literature to suggest these genes belong in the corresponding categories. **Column 10** of the grid indicates whether the encoded proteins are predicted to be secreted, according to the SignalP (S) or TargetP (T) prediction algorithms. Significantly down-regulated genes that could not be assigned a function are not shown in this figure.

To investigate the down-regulated genes from the *Neurospora grhl* AHC samples in more detail, we undertook a manual classification of these genes based on database and literature searches. We were especially interested in finding studies carried out directly on the *Neurospora crassa* genes or on their close homologs in other fungal species. Of the 83 significantly down-regulated genes, 54 had known functions, or predicted functions based on homology to genes in other fungi (Figure 3).

Strikingly, the most strongly down-regulated gene on the entire microarray (other than *grhl* itself) was *chitinase 1* (NCU04883) (Figure 3), the lack of which is likely to contribute to the conidial-separation phenotype observed in *grhl*/*csp-2* mutants. The *chitinase 1* gene of *Neurospora* has two consensus GRHL DNA binding sites (AAACGGTT & CACCGGTT) within 875 bp of the ATG codon for the *chitinase 1* gene. This suggests that the microarrays had identified at least some biologically relevant genes that are directly regulated by GRHL. There were at least six other GRHL-dependent down-regulated genes that encode proteins predicted (from research on other fungi) or known to be involved in normal “Cell Wall Structure” (Figure 3). All of these proteins contain predicted secretion signals, and four are experimentally verified components of the *Neurospora* cell wall: *gel1* (NCU07253, which has an AACCGGTT sequence ∼130 bp upstream of the transcription start), *Mwg1* (NCU05974, which has an AACCGGTT sequence ∼400 bp upstream of the 5′ end of the open reading frame), *non-anchored cell wall protein-5* (NCU00716, which has an AACAGGTT sequence ∼1.8 kb upstream of the transcription start), and *BYS1 domain-containing protein* (NCU08907) [46]. Three of the down-regulated cell wall genes – *Mwg1* and NCU04431 (both belonging to glycoside hydrolase family 16) as well as *gel1* (belonging to glycoside hydrolase family 72) – encode beta-1,3-glucanases. Beta-1,3-glucans are the major biopolymer constituent of the cell wall in filamentous fungi, and it has been shown in many fungal species that beta-1,3-glucanase enzymes are very abundant components of the cell wall, where they play an active role in cell wall biosynthesis and remodeling, as well as in processes such as biofilm formation [46]–[49]. For instance, mutations in enzymes from glycoside hydrolase family 72 cause cell wall defects in *S. cerevisiae* [48] and also affect morphogenesis and virulence in *Aspergillus fumigatus* [50]. We also found that seven of the 29 down-regulated genes that could not be assigned a function encode proteins with predicted secretion signals, and therefore might be components of the cell wall (Figure 3).

We classified 15 *grhl*-dependent down-regulated genes in the category “Virulence/Defense/Detoxification” (Figure 3). Defense/Virulence have not been studied experimentally in *Neurospora crassa*, as its normal host-pathogen relationships are unknown, so the functions of the following genes are inferred from research in other fungi. Genes potentially involved in defense include the following: *kynureninase* (NCU09183, which has two ACAGGTT sites ∼ 650 bp upstream of the open reading frame) and *indoleamine 2,3-dioxygenase* (NCU01402), which are involved in tryptophan catabolism and microbial growth control [43] (see above); NCU05495 encodes a putative anti-viral factor [51], [52]; and *exo-beta-1,3-glucanase* (NCU04850) is possibly involved in the degradation of foreign polysaccharides. Other *Neurospora grhl*-dependent genes potentially involved in fungal virulence include the following: the metalloprotease *MEP1* (NCU07200), whose homolog in *C. posadasii* has been shown to be crucial for evasion of host-detection [53]; the p450 monooxygenase *lovA* (NCU05376), whose homolog in a *Fusarium* species has been shown to be directly involved in mycotoxin synthesis [54]; *cerato-platanin* (NCU07787), which is potentially important for phytotoxin synthesis [55]; the integral membrane protein *pth11* (NCU06328), whose homolog in another fungal species is important for appressorium formation [56]; and NCU03643, which encodes a cutinase transcription factor that is likely to control plant cuticle digestion during fungal infection [57]. Finally, several genes potentially involved in the detoxification of harmful chemicals and the stress response include the following: the p450 gene *pisatin demethylase* (NCU06327), whose ortholog is important in fungal pea-pathogens for detoxifying host defensive chemicals [58]; the *aldehyde dehydrogenase* gene (NCU03415), which encodes a broadly acting detoxification and metabolic enzyme; the genes *catalase-3* (NCU00355) (another verified component of the cell wall [46]) and *NAD(P) transhydrogenase* (NCU01140), which encode enzymes known to be important for oxygen-radical detoxification; and the *YBH1 flavohemoglobin* gene (NCU10051), which may be involved in the stress response [59].

Taken together, these results suggest that *Neurospora* GRHL plays an important role in the regulation of genes that form and remodel the cell wall (at least in developing conidia). Additionally, a significant number of GRHL-dependent down-regulated genes in the aerial hyphae and conidia are involved in virulence, defense, and detoxification. It is should be noted that the cell wall and virulence/defense categories are not mutually exclusive, as seven of the 15 “defensive” gene products (e.g., *cerato-platanin* and *MEP1*) have secretion signals (Figure 3) and are likely to be deposited into the cell wall or released into the extracellular space.

### Microarray Profiling of Late-stage *Drosophila grh* Embryos

As a comparison to the *Neurospora* microarray dataset, we also carried out microarray-based transcriptome profiling of *Drosophila grh^IM^* and control wild-type embryos collected during late-stage 16 and early-stage 17 of embryogenesis [60], when cuticle deposition is occurring. We used flies homozygous for the *grh^IM^* allele because it is the strongest *grh* allele available (with respect to its cuticle and head-skeleton phenotypes) and because homozygous embryos do not produce any detectable GRH protein (assayed using an antibody against the C-terminal half of the protein [6]). By sequencing the *grh^IM^* transcript, we identified the lesion responsible for the *grh^IM^* allele as a TAT to TAA stop-codon introduction in exon seven, in the N-terminal end of the DNA-binding domain and about half-way through the protein (Figure 1B, amino acid Y29 in the *D.mel GRH* DNA-binding domain; see Figure S4 for details); this mutation is consistent with a functional null phenotype for *grh^IM^* and the lack of GRH protein detection with a C-terminal specific antibody [6]. See the Materials and Methods and Text S1 for more details on the *Drosophila* microarrays and data analyses. Besides the mutation in *grh*, the control and mutant embryos differed slightly with respect to their genetic backgrounds, as the wild-type strain contained the *yellow^1^* allele, which has an adult pigmentation defect, while the *grh^IM^* embryos were *yellow^+^*. However, we do not believe this significantly influenced our results since our microarray data indicate that *yellow* is expressed at extremely low levels during the embryonic stages we tested; furthermore, the *yellow* transcript expression levels were not significantly different between the *grh^IM^* and control embryos on the microarrays.

### Highly Enriched Gene Ontology Categories of the Misregulated Genes from the *Drosophila grh* Embryo Microarrays

Assayed at stages 16–17 of embryogenesis, zygotic loss of GRH function has a huge impact on the *Drosophila* transcriptome as a whole, as over 1,200 genes (FDR <0.01) were seen to be misregulated (up or down) in *grh^IM^* mutants compared with wild type (see the Materials and Methods section for the accession numbers of the *Drosophila* microarray datasets). Verification of microarray fold-change directionality for eight genes using quantitative RT-PCR is shown in Figure S5.

A search for enriched GO “Biological Process” (BP) and “Molecular Function” (MF) categories was performed (Text S1), and the top ten and eleven most significant classes, respectively, are shown in Table 1 (see Table S1 for the full lists of the significantly enriched GO-BP, GO-MF, and GO “Cellular Component” categories). As GRH is known to be very important for cuticle development and wound healing in *Drosophila*, we expected to see numerous genes involved in these processes misregulated on the microarrays. Indeed, four of the most significant GO-BP classes (e.g., “chitin metabolic process” and “aminoglycan metabolic process”) and five of the most significant GO-MF classes (e.g.,“structural constituent of the cuticle” and “chitin binding”) are consistent with the known role of GRH in regulating the formation of chitin-based cuticular barriers.

Surprisingly, the remaining six GO-BP categories (of the top ten) were all composed of genes involved in either innate immunity or the stress response (e.g., “defense response”, “immune response”, and “humoral immune response”). Similarly, the remaining six GO-MF categories (of the top eleven) were all composed of genes that encode products with either serine-protease or serine-protease-inhibitor activity. This was interesting because serine protease cascades are used to trigger the hemolymph melanization-reactions used in response to infection, and serine protease inhibitors (also known as serpins) are used to limit the spread of this reaction (for a review see [44]). This was initially puzzling, as most of these genes were seen to be up-regulated in the *grh^IM^* mutants (Table 2), and GRH has no known function as an inhibitor of the immune response.

**Table 2:** Select misregulated genes from the late-stage *grh^IM^* embryo microarrays.

Cuticle Formation/Chitin Metabolism (64)				
CG #	Gene Name or Symbol	Protein Type/*Process*	Fold (wt value)*	FDR
CG2044	Lcp4	cuticle protein	−139.41 (13854)*	2.36E−07
CG30163	Cpr60D	cuticle protein	−51.83 (5170)*	1.20E−06
CG18066	Cpr57A	cuticle protein	−32.87 (38041)	4.27E−06
CG15515	−	cuticle protein	−28.37 (75931)	7.15E−06
CG2043	Lcp3	cuticle protein	−14.34 (1067)*	3.64E−05
CG18140	Chitinase 3	*chitin metabolism*	−12.9 (2331)	2.48E−05
CG7941	Cpr67Fa1	cuticle protein	−11 (109921)	4.05E−05
CG6955	Lcp65Ad	cuticle protein	−9.9 (1824)	5.80E−05
CG4052	Cpr5C	cuticle protein	−8.72 (4115)	7.31E−05
CG8697	Lcp2	cuticle protein	−6.65 (422)*	1.60E−04
CG32400	Lcp65Ab1	cuticle protein	−6.64 (73278)	1.65E−04
CG17052	obstructor-A	*cuticle organization*	−5.67 (106803)	2.64E−04
CG8510	Cpr49Af	cuticle protein	−5.66 (5870)	2.75E−04
CG9070	Cpr47Eg	cuticle protein	−5.54 (11869)	2.79E−04
CG6217	knickkopf	*cuticle organization*	−5.29 (5171)	3.19E−04
CG14250	TweedleQ	cuticle protein/*body shape*	−5.09 (533)	3.72E−04
CG7287	Lcp65Aa	cuticle protein	−4.94 (1261)	3.91E−04
CG4778	obstructor-B	*cuticle organization*	−4.87 (16760)	3.88E−04
CG18773	Lcp65Ab2	cuticle protein	−4.45 (15581)	5.24E−04
CG7216	Acp1	cuticle protein	−4.43 (3406)	5.02E−04
CG14643	TweedleG	cuticle protein/*body shape*	−4.02 (117093)	6.66E−04
CG9369	miniature	*cuticle organization*	−3.77 (696)	1.33E−03
CG14639	TweedleF	cuticle protein/*body shape*	−3.32 (30236)	1.79E−03
CG10297	Acp65Aa	cuticle protein	−3.28 (4421)	1.38E−03
CG11650	Lcp1	cuticle protein	−3.23 (235)*	1.18E−03
CG10529	Lcp65Ae	cuticle protein	−3.17 (12315)	1.31E−03
CG5883	−	*chitin metabolism*	−3.06 (2218)	1.38E−03
CG7548	−	cuticle protein	−2.75 (8307)	2.01E−03
CG9535	mummy	*chitin biosynthesis*	−2.63 (43714)	2.37E−03
CG11142	obstructor-E	*cuticle organization*	−2.56 (6997)	2.77E−03
CG5494	Cpr92F	cuticle protein	−2.5 (39488)	2.92E−03
CG33302	Cpr31A	cuticle protein	−2.5 (142683)	2.95E−03
CG18779	Lcp65Ag3	cuticle protein	−2.46 (233203)	3.08E−03
CG12009	−	*chitin metabolism*	−2.4 (2092)	6.86E−03
CG7252	−	*chitin metabolism*	−2.24 (3566)	4.48E−03
CG9295	Cpr76Bc	cuticle protein	−2.2 (303)	4.82E−03
CG12755	l(3)mbn	cuticle protein	−2.18 (380)	4.95E−03
CG32499	Cda4	*chitin metabolism*	−2.13 (24891)	6.23E−03
CG15008	Cpr64Ac	cuticle protein	−2.09 (2006)	5.95E−03
CG18778	Cpr65Au	cuticle protein	−2.07 (534)	6.47E−03
CG32404	Cpr65Aw	cuticle protein	−2.03 (211)	6.89E−03
CG5812	TweedleT	*chitin metabolism*	−1.91 (27604)	9.30E−03
CG9781	obstructor-G	cuticle protein/*body shape*	1.78 (89)*	9.48E−03
CG9307	Chitinase 5	*cuticle organization*	1.78 (15030)	8.48E−03
CG9079	Cpr47Ea	*chitin metabolism*	1.84 (4076)	8.29E−03
CG8515	Cpr49Ah	cuticle protein	1.85 (78)*	6.72E−03
CG2555	Cpr11B	cuticle protein	1.9 (1069)	5.96E−03
CG6773	sec13	cuticle protein	1.95 (1358)	4.89E−03
CG9665	Cpr73D	*cuticle organization*	1.99 (1041)	6.30E−03
CG7876	Muc18B	cuticle protein	2.12 (5161)	3.78E−03
CG10725	−	*chitin metabolism*	2.22 (4127)	2.45E−03
CG7539	Edg91	cuticle protein	2.26 (20178)	2.19E−03
CG4784	Cpr72Ec	cuticle protein	2.29 (180)	2.77E−03
CG10533	Lcp65Af	cuticle protein	2.34 (61521)	2.70E−03
CG15006	Cpr64Aa	cuticle protein	2.46 (148)	1.48E−03
CG10531	Chitinase 9	*chitin metabolism*	2.46 (108)	1.43E−03
CG10140	−	*chitin metabolism*	2.84 (470)	7.52E−04
CG16963	Crystallin	cuticle protein; eye lens protein	3.42 (399)	3.81E−04
CG31080	TweedleH	cuticle protein/*body shape*	3.55 (8962)	4.30E−04
CG9076	Cpr47Ed	cuticle protein	3.91 (87)*	2.19E−04
CG13224	Cpr47Eb	cuticle protein	5.33 (4635)	8.76E−05
CG32284	−	*chitin metabolism*	5.57 (221)	7.66E−05
CG34271	Cpr65Ay	cuticle protein	9.56 (211)	1.61E−05
CG8836	Cpr49Ad	cuticle protein	35.79 (68)*	8.68E−07
**Melanization/Wound Healing (9)**				
**CG #**	**Gene Name or Symbol**	**Protein Type/** ***Process***	Fold (wt value)*	**FDR**
CG10501	a methyl dopa-resistant	*dopamine synthesis*	−5.46 (15116)	3.05E−04
CG1963	Pcd	*dopamine synthesis*	−2.92 (6569)	1.65E−03
CG42639	prophenol oxidase A1	*melanization effector*	−2.29 (14798)	4.14E−03
CG10244	Cad96Ca/Stitcher	atypical RTK/*wound healing*	−1.91 (12245)	9.35E−03
CG1102	MP1	serine protease/*melanization activator*	1.75 (4362)	9.94E−03
CG15825	fondue	*hemolymph coagulation*	2.02 (23174)	4.32E−03
CG3066	MP2/Sp7/PAE1	serine protease/melanization activator	2.53 (1283)	1.27E−03
CG1689	lozenge	*crystal cell differentiation*	3.32 (100)*	3.91E−04
CG10118	pale	tyrosine hydroxylase/*dopamine synthesis*	3.77 (6615)	2.53E−04
**Serine Proteases and Serpins (44)**				
**CG #**	**Gene Name or Symbol**	**Protein Type/** ***Process***	Fold (wt value)*	**FDR**
CG11912	−	serine protease {6}	−42.33 (2411)*	2.37E−06
CG7722	Spn47C	serpin	−22.9 (1389)*	8.99E−06
CG16997	−	serine protease {2}	−6.98 (9899)	1.38E−04
CG16704	−	serpin {2,6}	−5.39 (973)	2.94E−04
CG1342	Spn100A	serpin	−5.39 (18723)	2.94E−04
CG4386	−	serine protease	−4.35 (1386)	7.19E−04
CG31200	−	serine protease {2}	−4.05 (593)	6.74E−04
CG11843	−	serine protease {2}	−3.11 (212)*	1.63E−03
CG2071	Ser6	serine protease	−2.66 (8292)	2.29E−03
CG12172	Spn43Aa	serpin	−2.4 (3524)	3.63E−03
CG12385	thetaTry	Trypsin	−2.36 (1322)	3.73E−03
CG18477	−	serine protease {6}	−2.17 (234)	5.14E−03
CG33160	−	serine protease	−1.99 (59006)	7.50E−03
CG6483	Jonah 65Aiii	serine protease {5,6}	−1.89 (29930)	9.49E−03
CG33127	−	serine protease {6}	1.81 (9020)	7.85E−03
CG5246	−	serine protease {2,6}	1.96 (77)*	6.13E−03
CG9649	−	serine protease {2}	2.01 (553)	4.25E−03
CG12388	kappaTry	Trypsin	2.04 (2297)	4.13E−03
CG3513	−	serpin	2.08 (151)	3.44E−03
CG9456	Spn1	serpin	2.14 (422)	4.06E−03
CG33329	Sp212	serine protease	2.15 (424)	3.84E−03
CG3344	−	serine protease {6}	2.19 (4432)	2.52E−03
CG5639	−	serpin	2.36 (8777)	1.79E−03
CG8869	Jonah 25Bii	serine protease {1,3,5,6}	2.4 (569)	1.62E−03
CG8871	Jonah 25Biii	serine protease {1,5}	2.51 (4743)	1.43E−03
CG9672	−	serine protease	2.54 (292)	1.40E−03
CG7754	iotaTry	Trypsin	2.57 (1749)	1.26E−03
CG18180	−	serine protease {1,5}	2.63 (65)*	1.07E−03
CG1859	Spn43Ad	serpin {1,2}	2.79 (3765)	8.27E−04
CG18681	epsilonTry	Trypsin {6}	2.96 (122)	6.83E−04
CG4998	−	serine protease	2.97 (66250)	6.21E−04
CG11668	−	serine protease	2.98 (282)	6.19E−04
CG11911	−	serine protease {2}	3.12 (20537)	5.85E−04
CG7432	−	serine protease	3.15 (3722)	4.96E−04
CG17571	−	serine protease {5}	3.59 (1111)	3.56E−04
CG4927	−	serine protease	3.68 (568)	2.96E−04
CG2045	Ser7	serine protease {1,2}	3.7 (146)	2.80E−04
CG12351	deltaTry	Trypsin	3.93 (355)	6.21E−04
CG33459	−	serine protease	6.68 (84)*	4.15E−05
CG30028	gammaTry	Trypsin	6.98 (181)	4.31E−05
CG8867	Jonah 25Bi	serine protease {3,5}	8.72 (109)	2.17E−05
CG9733	−	serine protease	9.14 (727)	1.81E−05
CG18211	betaTry	Trypsin	24.12 (106)	2.13E−06
CG4821	Tequila	Trypsin; Neurotrypsin ortholog {6}	46.32 (731)	5.32E−07
**Innate Immunity (37)**				
**CG #**	**Gene Name or Symbol**	**Protein Type/** ***Process***	Fold (wt value)*	**FDR**
CG18108	IM1	putative AMP {1,2,6}	−58.95 (3622)*	9.45E−07
CG14823	−	lysozyme	−9.02 (6023)	6.43E−05
CG7709	Mucin 91C	ECM component	−2.63 (46409)	2.48E−03
CG7106	lectin-28C	putative PRR	−2.31 (420)	4.25E−03
CG30062	−	lysozyme	−2.05 (293)	6.60E−03
CG6124	eater	PRR/*phagocytosis*	−1.94 (356)	8.79E−03
CG1179	LysB	lysozyme	1.84 (382)	6.80E−03
CG5008	GNBP3	PRR (Fungi)/*Toll-signaling*	1.96 (189)	5.55E−03
CG18279	IM10	putative AMP {1,6}	1.97 (6532)	6.84E−03
CG6426	−	lysozyme	2.02 (26238)	4.31E−03
CG10146	Attacin-A	AMP (GN Bacteria) {1,2,3,4,6}	2.02 (80)*	5.19E−03
CG16705	SPE	serine protease/*Toll-signaling*	2.06 (3498)	4.04E−03
CG7876	Mucin 18B	ECM component	2.12 (5161)	3.78E−03
CG14704	PGRP-LB	catalytic PGRP {1,6}	2.13 (240)	2.97E−03
CG11159	−	lysozyme	2.24 (326)	2.52E−03
CG1180	LysE	lysozyme	2.44 (439)	1.50E−03
CG33717	PGRP-LD	PRR	2.63 (1653)	1.08E−03
CG4432	PGRP-LC	PRR (GN Bacteria)/Imd-signaling {1}	2.63 (474)	1.06E−03
CG15678	pirk	response to symbiotic bacteria	2.87 (998)	7.21E−04
CG9697	PGRP-SB2	catalytic PGRP	3.12 (67)*	5.07E−04
CG8175	Metchnikowin	AMP (Fungi) {1,2,3,6}	3.3 (296)	4.05E−04
CG15065	IM2-like	putative AMP {1,2}	4.01 (1102)	1.95E−04
CG1165	LysS	lysozyme	4.17 (204)	1.70E−04
CG10794	Diptericin B	AMP (GN Bacteria) {1,3,4}	4.19 (193)	1.70E−04
CG15231	IM4	putative AMP {1,6}	4.35 (13040)	1.88E−04
CG16844	IM3	putative AMP {1,3,6}	5.45 (10195)	7.45E−05
CG32279	drosomycin-2	AMP (Fungi)	5.49 (177)	7.38E−05
CG15066	IM23	putative AMP {1,6}	5.69 (853)	7.10E−05
CG9120	LysX	lysozyme	5.82 (74)*	6.24E−05
CG18372	Attacin-B	AMP (GN Bacteria) {1,2,3,4,6}	6.44 (77)*	5.62E−05
CG10810	Drosomycin	AMP (Fungi) {1,2,3}	6.6 (1095)	4.31E−05
CG4740	Attacin-C	AMP (GN Bacteria) {1,3,4}	6.63 (72)*	4.31E−05
CG13422	−	PRR {1,2,6}	7.12 (64)*	3.36E−05
CG18106	IM2	putative AMP {1,2,3,6}	7.95 (3140)	3.45E−05
CG2958	lectin-24Db	putative PRR	9.12 (75)*	1.67E−05
CG9118	LysD	lysozyme	11.69 (319)	7.15E−06
CG10812	drosomycin-5	AMP (Fungi) {1,2}	138 (81)*	1.33E−07
**Cytoskeleton/Cell Adhesion/Apico-Basal Polarity (19)**				
**CG #**	**Gene Name or Symbol**	**Protein Type/** ***Process***	Fold (wt value)*	**FDR**
CG9379	blistery	tensin/*focal adhesion component*	−6.28 (13823)	1.91E−04
CG31190	Dscam3	*homophilic cell adhesion*	−6.21 (455)*	2.03E−04
CG18250	Dystroglycan	*apico-basal polarity; anchoring to ECM*	−4.81 (862)	4.49E−04
CG31009	Cad99C	cadherin/*actin organization*	−4.01 (6509)	6.87E−04
CG42610	Fhos	*actin organization*	−3.67 (11613)	9.04E−04
CG3320	Rab1	small GTPase/*actin organization*	−2.57 (15615)	2.56E−03
CG6445	Cad74A	cadherin/*cell adhesion*	−2.36 (2895)	4.05E−03
CG5055	bazooka	Par3 homolog/*apico-basal polarity*	−2.31 (2298)	3.96E−03
CG17716	faint sausage	*epithelial morphogenesis*	−2.29 (7220)	4.87E−03
CG42734	Ankyrin 2	*microtubule organization*	−1.98 (2520)	7.70E−03
CG12437	raw	*epithelial morphogenesis*	1.75 (2601)	9.96E−03
CG42614	scribbled	*apico-basal polarity*	1.83 (2482)	7.13E−03
CG17957	Sry-alpha	*actin organization*	1.87 (215)	6.86E−03
CG6976	Myo28B1	myosin/*molecular motor*	1.9 (1697)	5.60E−03
CG4316	Stubble	serine protease/*actin organization*	2.07 (587)	4.89E−03
CG33979	capulet	*actin organization*	2.13 (818)	3.60E−03
CG10125	zero population growth	gap junction channel	2.21 (127)	2.63E−03
CG8978	Suppressor of profilin 2	*actin organization*	2.26 (13355)	2.34E−03
CG5178	Act88F	actin	2.43 (95)*	1.53E−03
**Detoxification (44)**				
**CG #**	**Gene Name or Symbol**	**Protein Type/** ***Process***	Fold (wt value)*	**FDR**
CG1944	Cyp4p2	P450 (Fat Body {7})	−87.19 (4690)*	4.20E−07
CG10241	Cyp6a17	P450 (Hindgut {7})	−56.78 (3284)*	8.68E−07
CG33503	Cyp12d1-d	P450 (Fat Body, Midgut, Malphigian Tubes {7}){8}	−22.22 (1314)*	9.99E−06
CG18730	Amylase proximal	detoxification {8}	−11.54 (1153)	3.31E−05
CG10842	Cyp4p1	P450 (Midgut, Malphigian Tubes {7})	−10.72 (5972)	4.31E−05
CG33546	gfzf	glutathione S-transferase	−10.04 (12617)	4.58E−05
CG17876	Amylase distal	detoxification {8}	−5.86 (885)	2.39E−04
CG9363	−	glutathione S-transferase	−5.49 (6388)	2.91E−04
CG1488	Cyp311a1	P450 (Midgut {7})	−4.11 (454)	6.29E−04
CG30489	Cyp12d1-p	P450 (Fat Body, Midgut, Malphigian Tubes {7})	−4.02 (1004)	7.24E−04
CG8652	Ugt37c1	glucuronosyltransferase	−2.56 (1005)	2.70E−03
CG9362	−	glutathione S-transferase	−2.33 (2238)	3.84E−03
CG31002	−	glucuronosyltransferase	−2.27 (1530)	4.29E−03
CG17527	GstE5	glutathione S-transferase	−2.22 (3038)	6.11E−03
CG12242	GstD5	glutathione S-transferase	−2.21 (223)	4.83E−03
CG13271	Ugt36Bb	glucuronosyltransferase	−2.19 (240)	4.97E−03
CG17525	GstE4	glutathione S-transferase	−2.16 (1686)	5.26E−03
CG5137	Cyp312a1	P450 (Gonads) {7}	−2.11 (322)	5.94E−03
CG11289	−	glucuronosyltransferase	−2.05 (997)	6.70E−03
CG8453	Cyp6g1	P450 (Fat Body, Midgut, Malphigian Tubes {7})	−2.03 (463)	7.11E−03
CG4688	−	glutathione S-transferase	1.78 (245)	8.52E−03
CG4026	IP3K1	oxidative stress response	1.8 (2853)	8.64E−03
CG1829	Cyp6v1	P450 (Gonads {7})	1.8 (97)*	8.38E−03
CG8587	Cyp301a1	P450 (Hindgut {7})	1.82 (7246)	7.51E−03
CG4772	Ugt86Dh	glucuronosyltransferase	1.82 (2621)	7.46E−03
CG6633	Ugt86Dd	glucuronosyltransferase {8}	1.85 (591)	9.71E−03
CG4381	GstD3	glutathione S-transferase	1.89 (537)	6.69E−03
CG10248	Cyp6a8	P450 (Malphigian Tubes {7}){8}	1.89 (2545)	9.56E−03
CG17534	GstE9	glutathione S-transferase	1.95 (4213)	5.17E−03
CG10240	Cyp6a22	P450 (Gonads {7})	1.96 (951)	4.99E−03
CG15102	Jheh2	detoxification {8}	1.99 (2525)	4.67E−03
CG15661	−	glucuronosyltransferase	2.01 (846)	6.75E−03
CG3943	kraken	digestion; detoxification	2.05 (8581)	4.02E−03
CG4485	Cyp9b1	P450 {7}	2.06 (533)	3.49E−03
CG5999	−	glucuronosyltransferase	2.28 (64)*	2.05E−03
CG1702	−	glutathione S-transferase	2.49 (2822)	1.49E−03
CG13270	Ugt36Ba	glucuronosyltransferase	2.73 (6015)	9.76E−04
CG11012	Ugt37a1	glucuronosyltransferase	4.47 (93)*	1.37E−04
CG3481	Adh	alcohol dehydrogenase	4.76 (21147)	1.21E−04
CG10245	Cyp6a20	P450 {7}	5.85 (2512)	6.56E−05
CG4302	−	glucuronosyltransferase	6.55 (2304)	4.20E−05
CG5724	−	glucuronosyltransferase {8}	9.13 (117)	1.74E−05
CG8345	Cyp6w1	P450 (Fat Body, Midgut, Malphigian Tubes {7}){8}	9.13 (128)	1.67E−05
CG18559	Cyp309a2	P450 (Gonads {7})	28.2 (94)*	1.58E−06
**Defense/Stress Response (18)**				
**CG #**	**Gene Name or Symbol**	**Protein Type/** ***Process***	Fold (wt value)*	**FDR**
CG32475	methuselah-like 8	GPCR	−44.54 (2412)*	1.68E−06
CG6530	methuselah-like 3	GPCR	−5.91 (1075)	2.36E−04
CG16954	Hsp60D	heat shock protein	−5.85 (546)*	2.42E−04
CG33117	Victoria	Turandot-like	−3.99 (500)	7.06E−04
CG2830	Hsp60B	heat shock protein	−3.06 (6325)	1.97E−03
CG4604	Glial Lazarillo	ApoD ortholog	−2.78 (9228)	1.94E−03
CG12002	Peroxidasin	ECM peroxidase {1,2,6}/ROS metabolism	−2.62 (6843)	2.91E−03
CG6646	DJ-1alpha	oxidative stress response	−2.5 (686)	3.29E−03
CG7052	TepII	opsonization; humoral response {1,2,6}	−2.19 (6648)	5.88E−03
CG6871	Catalase	ROS metabolism; hydrogen peroxide breakdown	2.25 (19730)	2.44E−03
CG31509	Turandot A	humoral stress response {6}	2.51 (68)*	1.34E−03
CG6186	Transferrin 1	Iron sequestration {2}	2.92 (105)	6.96E−04
CG4183	Hsp26	heat shock protein {1}	3.76 (563)	2.47E−04
CG6489	Hsp70Bc	heat shock protein {1,3,4}	4.75 (156)	1.11E−04
CG31449	Hsp70Ba/Bb/Bbb	heat shock protein {4}	5.51 (297)	7.73E−05
CG31508	Turandot C	humoral stress response {6}	5.54 (70)*	7.52E−05
CG31366	Hsp70Aa/Ab	heat shock protein {4}	7.48 (1513)	3.13E−05
CG31359	Hsp70Bb/Bbb	heat shock protein {4}	7.54 (383)	2.69E−05

Select significantly misregulated genes were manually classified in into the following categories: Cuticle Formation/Chitin Metabolism; Melanization/Wound Healing; Serine Proteases/Serpins; Innate Immunity; Cytoskeleton/Cell Adhesion/Apico-Basal Polarity; Detoxification; and Defense/Stress Response. **“CG #”** refers to the accession numbers from FlyBase. **“Gene Name or Symbol”** refers to either the full gene name or the gene symbol on Flybase; this column is blank if no assigned gene name was found in FlyBase. **“Protein Type/** ***Process*** **”** refers to experimentally verified or putative (most often based on homology) functions assigned to the genes. Numbers in curly brackets refer to studies in which these genes were also seen to be misregulated upon the following treatments: {1} bacterial infection [87]–[89]; {2} fungal infection [87], [88], [90]; {3} viral infection [90], [91]; {4} *Wolbachia* infection [90], [92]; {5} Microsporidia infection [90]; and {6} parasitoid infection [93], [94]. Categories {1–6} were adapted from [66]. {7} refers a systematic analysis of the expression patterns of the *Drosophila* p450 genes [68].{8} refers to a systematic analysis of detoxification genes in *Drosophila* [69]. “Fold (wt value)*” refers to the fold changes seen in the expression of these genes relative to wild type. Absolute wild-type fluorescence values are shown in parentheses. An asterisk next to a value means the lowest value in the *grh*/WT ratio was near baseline (∼100 units of fluorescence), which could artificially inflate the fold-change values. **“FDR”** refers to the False Discovery Rates calculated for each gene. All genes shown have an FDR value of less than 0.01.

From these we results, we conclude that in addition to the expected misregulation of genes involved in cuticle formation, late-stage *grh^IM^* embryos are experiencing a massive wound/immune response as well. During the stages they were collected (late-stage 16 or early-stage 17 of embryogenesis) the *grh^IM^* embryos have weaker and more permeable epidermal barriers [1], [6], [12], yet are still motile, which can cause their fragile cuticles to rupture. Consistent with this, the *pale* gene, which encodes tyrosine hydroxylase, is known to be up-regulated around sterile wound sites in a largely *grh*-independent manner [3], [4], and in *grh^IM^* embryos, *pale* transcripts are significantly up-regulated. This is also consistent with the observation that clean puncture wounding of late-stage embryos (in the absence of intentional microbial infection) also induces the expression of large numbers of *Drosophila* genes involved in innate immunity and the stress response (R. Patterson & W. McGinnis, unpublished).

### Misregulated Genes from the *Drosophila grh* Embryo Microarrays Reflect the Role of GRH in Barrier Formation and Wound Healing

We carried out a manual classification of the genes both and up- and down-regulated on the *Drosophila grh^IM^* embryo microarrays, and select genes are shown in Table 2. We placed 64 genes in the category “Cuticle Formation/Chitin Metabolism”, including genes involved in the generation and degradation of chitin molecules, as well as genes for many cuticle proteins that are deposited into the cuticle to mediate aspects of cuticle-shape and elasticity [61], [62]. The majority of these genes (42 of 64) were down-regulated, consistent with potential direct regulation by GRH, and consistent with the idea that GRH is a crucial regulator of physical-barrier formation in *Drosophila*. However, a subset of these "cuticle/chitin" genes (22 of 64) were seen to be up-regulated in the *grh* mutants. It is possible that these up-regulated cuticle genes are normally directly repressed by GRH in wild-type embryos, but we believe it more likely that they are being overexpressed to compensate for the lack of the GRH-activated cuticle proteins, or they are being overexpressed in response to cuticular damage in *grh^IM^* mutants (see above).

Interestingly, one of the most strongly down-regulated genes on the microarray was *chitinase 3* (∼13 fold down) (Table 2), which is a *Drosophila* homolog of *Neurospora chitinase 1*– the most strongly down-regulated gene in the *Neurospora grhl* mutants (see above). We identified three high-affinity GRH binding sites within the two kb region upstream of the *Drosophila chitinase 3* transcriptional start site, and *chitinase 3* is also extensively co-expressed with GRH throughout the *Drosophila* epidermis and tracheal system during embryogenesis (data not shown), consistent with direct regulation by GRH.

Also consistent with the *grh* mutant phenotypes, 19 genes known or predicted to be involved in a category we called “Cell Adhesion/Apical-Basal Polarity/Cytoskeleton” were significantly misregulated (Table 2). This category includes three Cadherin-domain-containing protein genes, including *Dystroglycan* (∼5 fold down), which is required for maintaining the apical-basal polarity of epithelial cells and anchoring the intracellular actin cytoskeleton to the extracellular matrix [63]. Another gene required for apical-basal polarity and adhesion of epidermal cells that was significantly down-regulated in *grh^IM^* mutants (∼2.3 fold down) is *bazooka* [64]. Previous studies by Narasimha et al. [13] have shown that two genes encoding components of the *Drosophila* septate junction – *coracle* and *Fasciclin 3*– were expressed at reduced levels in *grh* mutant clones in imaginal disc epithelia, and that *coracle* and *Fasciclin 3* gene expression could be activated by ectopic GRH protein in embryonic amnioserosa cells. As measured by our late-stage embryonic *grh* mutant microarrays, *coracle* transcript levels were very slightly lowered, but the difference compared to wild-type levels was not statistically significant. *Fasciclin 3*, which was probed by eleven different sequences on the microarray chip we used, was reproducibly reduced in *grh* mutants; we found that the expression of this gene was ∼25% lower than wild type in every experimental and biological replicate, although this difference was never significant enough to reach the stringent FDR threshold we set. Therefore, it is important to note that since we used whole embryos as the RNA source for our microarray experiments there are likely to be many true GRH-regulated genes that were not identified as significant in our analyses, such as coracle and *Fasciclin 3*. It is likely that this category will include genes whose expression is only quantitatively changed in *grh* mutant backgrounds, or whose expression is limited to only a subset of the cells that produce GRH protein, and thus would not pass our FDR threshold.

We placed nine genes in the category “Melanization/Wound Healing” (Table 2), four of which were down-regulated. The three most strongly down-regulated genes in this class (*alpha methyl dopa-resistant*, *Pcd*, and *prophenol oxidase A1*) are known or suspected to be directly involved in the cuticular melanization/sclerotization pathway. *Stitcher/Cad96Ca*, a wound-induced gene known to be directly activated by GRH [5], was significantly down-regulated on our microarrays. *Dopa decarboxylase*, another gene known to be directly dependent on GRH for its expression during development [1] and the epidermal wound-response [3], was down-regulated (∼1.7 fold down), although it did not pass the stringent FDR threshold we set and is not shown in Table 2.

### Misregulated Genes from the *Drosophila* Microarrays Indicate Mutation of *grh* Triggers Innate-immune and Stress-response Pathways

While a comprehensive analysis of all the genes involved in innate immunity, stress, and detoxification that were seen to be misregulated in *grh^IM^* mutants is beyond the scope and focus of this paper (and because many are very likely to be misregulated due to indirect effects of the *grh^IM^* phenotype), we will only briefly review the major classes of genes.

Thirty-seven genes in the category “Innate Immunity” (Table 2) were misregulated in *grh^IM^* embryos, and they included genes from nearly every aspect of *Drosophila* innate immunity [44]. One innate-immune gene, *IM1*, was strongly down-regulated, suggesting the potential for direct activation of *IM1* by GRH in the epidermis. Consistent with this possibility, there is a near perfect palindromic GRH binding site (AACTGGTTT) found less than 600 bp upstream of the *IM1* gene. Other down-regulated immunity genes potentially under the direct control of GRH include *lectin-28C*, *Mucin 91C*, *eater,* and two putative lysozymes. However, the majority of innate-immune genes that were misregulated in *grh^IM^* mutants were up-regulated (31 of 37), and they include known antimicrobial peptides (e.g., *Attacins*, *Drosomycins*, *Diptericin B*, and *Metchnikowin*), lysozymes, Pattern-Recognition Receptors, and the Toll-signaling activator *Spaetzle-Processing Enzyme* (*SPE*).

Eighteen genes in the “Defense/Stress Response” category were misregulated in *grh^IM^* embryos (Table 2). The two most strongly down-regulated genes were *methuselah-like 8* and *3*. Mutations in a paralogous gene (*methuselah*) have been correlated with longer lifespan and increased resistance to stress in *Drosophila* [65], so it possible that the observed down-regulation of *mthl-8* and *-3* was a response to tissue damage and stress in these embryos. Seven heat shock protein (hsp) genes were also misregulated in *grh^IM^* embryos, five of which were up-regulated and have been shown elsewhere to be differentially expressed upon infection of adult flies with microbes (*Hsp26*, *70Bc*, *70Bb*, *70Bbb*, and *70Aa*) [66]. *Turandot A*, *C*, and *Victoria* were also seen to be up-regulated, which are believed to act as extra-cellular chaperones, binding to denatured proteins in the hemolymph that are released upon tissue damage or stress [67].

Forty-four genes in the “Detoxification” category were misregulated in *grh^IM^* embryos (Table 2). These include multiple cytochrome p450 genes [68] as well as glutathione S-transferases and UDP-glucuronosyltransferases, which function by chemically modifying toxic compounds in the cytoplasm or hemolymph in order to render them less active [69]. The misregulation of these genes (which were both up- and down-regulated in nearly equal proportions) is still somewhat unclear, although we propose that their expression levels are altered in response to the release of toxic endogenous compounds during tissue damage in *grh^IM^* mutants.

Taken together, these results indicate that late-stage *grh^IM^* embryos have reduced expression of a wide variety of extracellular cuticular-barrier genes as well as a number of cell-cell adhesion genes [13] (Tables 1, 2, and S1); in addition they are experiencing a massive wound/immune response and are undergoing extreme stress, likely due to global tissue damage in response to cuticular tearing or epidermal-barrier permeability [6].

### GRH is Required for Epidermal Integrity During Larval Stages of *Drosophila* Development

**Figure 4:**
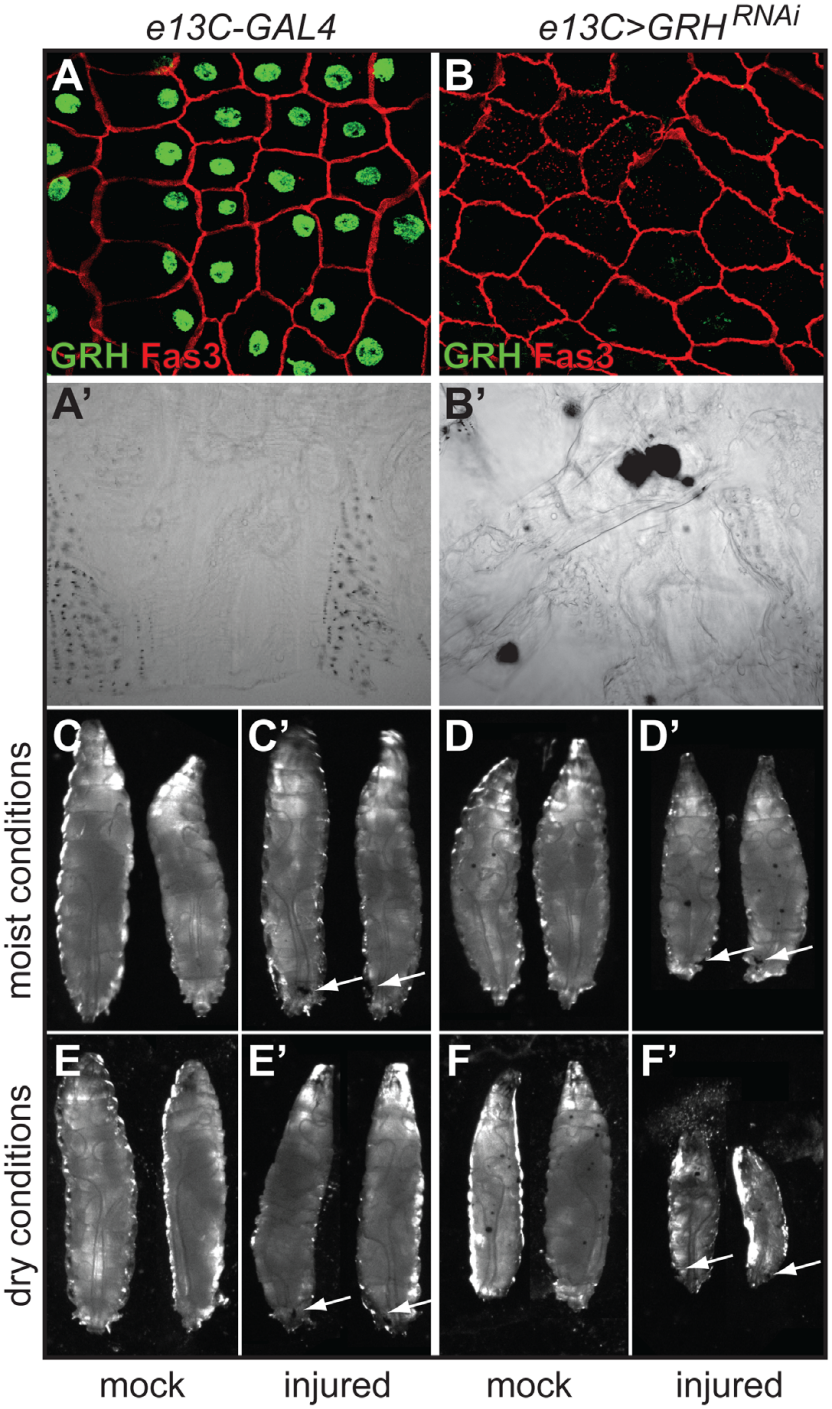
Knock-down of GRH in the larval epidermis leads to dramatically increased fluid loss following injury. (**A** and **B**) Whole-mount preparations of dissected larval epidermal tissue from 5 day old control larvae (*e13C-GAL4*) (A) or 5 day old larvae expressing a *UAS-GRH^RNAi^* transgene driven by *e13C-GAL4* (*e13C>GRH^RNAi^*) (B). Antibody stains for GRH (green) and Fasciclin 3 (red) are shown. (**A**’ and **B**’) Close-ups of cuticle preparations of dissected larval epidermises from 5-day-old (at the wandering stage, just prior to puparium formation) control larvae (*e13C-GAL4*) (A’) or 5-day-old larvae expressing a *UAS-GRH^RNAi^* transgene driven by *e13C-GAL4* (*e13C>GRH^RNAi^*) (B’). The large dark spots seen in B’ are localized depositions of melanin that appear at random positions in the epidermis/cuticle when GRH is knocked down during larval stages. (**C–F**’) Control larvae (*e13C-GAL4*) and GRH knock-down larvae (*e13C>GRH^RNAi^*) were left untreated (C, D, E, and F) or punctured with a clean needle (injured; C’, D’, E’, and F’) under moist (C, C’, D, and D’) or dry (E, E’, F, and F’) conditions. Sites of injury are indicated with arrows.

We wished to more fully characterize the phenotypes of *grh* null mutants at later stages of the *Drosophila* life cycle. However, as *grh* null-mutant embryos die at the embryonic/larval transition, this prohibited us from determining the function of GRH in *grh^IM^* larvae and adults. To circumvent this, we produced a *Drosophila* strain in which GRH is knocked down in the larval epidermis (*e13C>GRH^RNAi^*) by crossing a transgenic *UAS-GRH^RNAi^* line with a strain containing the larval driver *e13C-GAL4*, which produces GAL4 in the larval epidermis, fat body, gut, imaginal discs, and salivary glands [70]. By immunostaining, we observed that GRH protein was undetectable in the epidermal tissues of late third-instar *e13C>GRH^RNAi^* larvae (Figure 4B) compared with control *e13C-GAL4* larvae (Figure 4A), demonstrating that RNAi-mediated knockdown of GRH is very efficient in third instar larvae. In both samples, epidermal cell boundaries are clearly marked by Fasciclin 3 staining, indicating that gross cellular morphology remains intact. It is possible that GRH is only partially knocked down during larval molts, allowing for the deposition of a cuticular barrier sufficient for larval survival, albeit a cuticular barrier that is defective enough to be more easily wounded and water-permeable in wandering third-instar larvae (see below).

While generally healthy, wandering third-instar *e13C>GRH^RNAi^* larvae all developed melanized clots of diverse size and distribution, which were never observed in control larvae (Figures 4A’ and B’) or in *e13C>GRH^RNAi^* larvae during the earlier first, second, or foraging third-instar stages (data not shown). Upon dissection it was found that these melanotic spots were tightly associated with epidermal cells of the body wall, similar to the phenotypes seen when both Dorsal and Dif transcription factors are knocked down during larval stages [70]. Furthermore, all *e13C>GRH^RNAi^* larvae died at the prepupal stage with noticeably decreased body size compared with wild type, despite the fact that experimental and control larvae appeared similar in size prior to wandering (data not shown).

A lack of GRH function during *Drosophila* embryogenesis leads to a fragile cuticle [1], [12] and increased epidermal permeability [6]. Therefore, it seemed likely that knocking down GRH during larval stages would have similar effects, which could explain both the presence of randomly localized melanized spots as well as the decreased body size (presumably due to fluid loss). In *Drosophila,* melanized spots have been associated with hyperactivation of the immune system [71], loss of both Dorsal and Dif transcription factors [70], and such dark spots also appear at wound sites, apparently to strengthen clots and prevent body-fluid loss following physical injury.

We propose that upon leaving the moist food source, wandering *e13C>GRH^RNAi^* larvae develop multiple “micro-wounds” (as evidenced by the ectopic melanotic spots) due to the fragility of their cuticles, which is exacerbated by the dry conditions on the vial walls. Furthermore, we hypothesized that the decreased body size observed in these larvae is a result of fluid loss due to increased epithelial permeability as well as a loss of hemolymph following micro-wounding. These observations suggest that during larval stages, GRH is required for the maintenance of epidermal/cuticular-barrier integrity.

To determine whether epidermal GRH activity is required in larvae to prevent body-fluid loss following wounding, we wounded wandering third-instar larvae with a sterile needle and let them recover in either moist or dry conditions. Under moist conditions, both control and *e13C>GRH^RNAi^* larvae maintained approximately the same body mass, even after clean injury (compare Figures 4C’ and D’). However, when injured *e13C>GRH^RNAi^* larvae were placed in dry conditions, they showed an obviously decreased body size 7 h after injury (Figures 4F’). Injured control, uninjured control, or uninjured *e13C>GRH^RNAi^* larvae did not show any obvious decreases in body size under dry conditions after 7 h (Figures 4E, E’, F). These results suggest that GRH activity in the epidermis is needed for properly repairing wounds and preventing catastrophic body-fluid loss following wounding under dry conditions. These effects could be due to failures or delays in wound healing in the knockout larvae, possibly caused by weakened cuticles due to lower GRH protein levels during larval stages.

We were curious to see if epidermal GRH knockdown could lead to desiccation in the absence of injury after longer periods (>7 h) in dry conditions. We incubated both injured and uninjured control and *e13C>GRH^RNAi^* larvae for ∼24 h under dry conditions. Although most uninjured (96.2±0.6%) and injured (65.6±3.9%) control larvae reached the prepupal stage without any obvious decreases in body size, all uninjured and injured *e13C>GRH^RNAi^* larvae (100%) showed decreases in body size and died before initiating the pupariation process (data not shown). These results indicate that the larval function of GRH is crucial for avoiding excessive body-fluid loss under dry conditions, and it is necessary for viability even in the absence of major injury.

### Silencing of GRH in Adult *Drosophila* Increases their Susceptibility to Septic Injury

**Figure 5:**
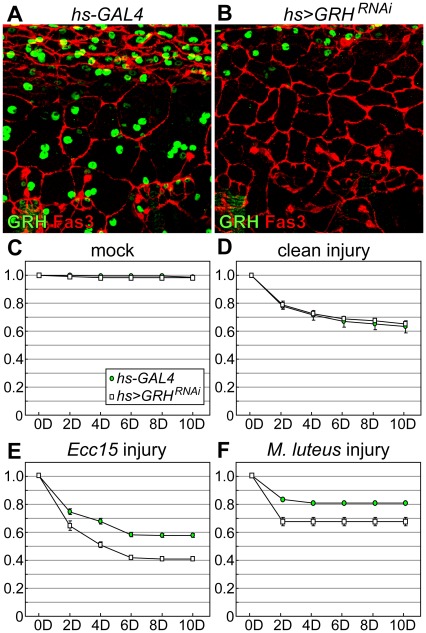
Loss of GRH function in adult flies increases their susceptibility to bacterial infection. (**A** and **B**) Whole-mount preparations of dissected adult abdominal epidermal tissue from control flies (*hs-GAL4*) and GRH knock-down flies expressing a *UAS-GRH^RNAi^* transgene driven by *hs-GAL4* (*hs>GRH^RNAi^*). Antibody stains for GRH (green) and Fasciclin 3 (red) are shown. (**C–F**) Survival curves from *hs-GAL4* and *hs>GRH^RNAi^* adults after mock treatment (C), clean injury (D), injury with a needle coated with gram-negative *Ecc15* bacteria (E), or injury with a needle coated with gram-positive *M. luteus* bacteria (F). The survival of adult flies was measured over the 10 day period after injury. The average values of three independent experiments are shown along with the standard errors of the mean.

Due to the relatively short time-course of larval development, and the fact that *e13C>GRH^RNAi^* larvae do not develop past the prepupal stage, examining the role of epidermal GRH expression in larval microbial resistance is problematic. Therefore, we focused on analysis of clean or septic injury in *Drosophila* adults in which *grh* function was knocked down by heat shock induced RNAi. We found that heat-shock driven expression of a *UAS-GRH^RNAi^* (*hs>GRH^RNAi^*) can efficiently eliminate GRH protein in most cells of the adult epidermis compared with similarly treated control flies containing only the *hs-GAL4* construct (Figures 5A and B). These *hs>GRH^RNAi^* flies were completely viable and did not develop the drying phenotypes observed in *e13C>GRH^RNAi^* larvae.

The *hs>GRH^RNAi^* flies did not show any reduction in normal life span compared with control flies, indicating that GRH is not required for the homeostatic maintenance of adult viability, at least under laboratory conditions (Figure 5C, and data not shown). Next, we challenged knockdown and control flies with either clean or septic injury using *Ecc15* (a gram-negative bacterium) or *M. luteus* (a gram-positive bacterium), and their survival was monitored over a 10 day period. Although control and *hs-GRH^RNAi^* flies showed similar survival curves after clean injury (Figure 5D), *hs>GRH^RNAi^* flies showed decreased survival after *Ecc15* and *M. luteus* infection than controls. Ten days after septic injury with *Ecc15* or *M. luteus*, 40 and 67% of the GRH knockdown adults survived, respectively, compared with 57 and 80% of control adults that were heat shocked without *GRH^RNAi^* knockdown (Figures 5E and F). These results suggest that GRH plays some protective role in *Drosophila* adults following microbial infection. However, our embryonic microarrays indicate that *grh* is not required to activate the standard repertoire of antimicrobial peptide genes, so it seems unlikely that GRH is crucial for activating the canonical genes of the innate immune response in adults [44]. Therefore, the exact nature of the protective effects imparted by GRH during *Drosophila* adulthood remains to be identified.

## Discussion

Is there an ancestral connection between the transcriptional control of many fungal cell walls and animal epidermal extracellular barriers? This question is probably impossible to answer definitively, given the vast evolutionary distances between extant fungal and animal lineages and the loss of so many transitional states. However, because of the high-level conservation of GRH-family function in animal epidermal barrier formation, we believed that by studying the function of transcription factors related to the GRH family in the filamentous ascomycete fungus *Neurospora crassa* we might shed some light on this question. We find that with respect to several amino acid residues predicted to be important for DNA-binding specificity, fungal GRH-like proteins are more similar in sequence to the GRH family than to the LSF family of transcription factors. Consistent with this, we show that the *Neurospora* GRHL protein can bind to the same DNA consensus site as metazoan GRH-like proteins in vitro (albeit with a lower affinity). Therefore, we believe the last common ancestor of Fungi and Metazoa was likely to have contained at least one CP2 superfamily protein that was more related, both structurally and functionally, to existing animal GRH family proteins than to existing animal LSF family proteins.

**Figure 6:**
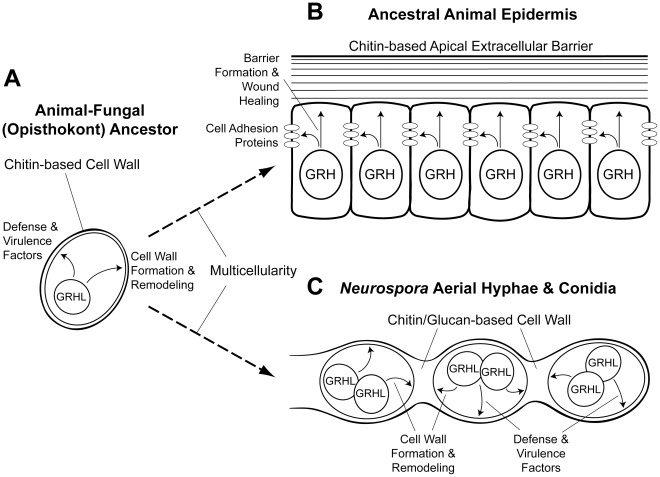
The proposed evolutionary functions of GRH-like transcription factors in the opisthokont lineage. (**A**) It is likely that the animal-fungal ancestor was a single-celled organism that possessed a chitin-based cell wall at some stages of its life cycle, and a flagellum at other stages (not shown). We propose that in this organism, a GRH-like transcription factor (GRHL) regulated aspects of physical-barrier formation and remodeling, for example, via the expression of enzymes such as chitinase. (**B**) In the lineage leading to animals, complex multicellular tissues were developed, including epithelia with chitin-based apical extracellular barriers (e.g., the ancestral arthropod epidermis). We believe it is possible that components of the ancestral opisthokont cell wall were repurposed (or redeployed) to form these chitin-based apical extracellular barriers, with GRLH proteins maintaining their role in barrier formation and remodeling during the process. An analogous process may have occurred during the evolution of multicellular volvocine algae [74]. (**C**) In the lineage leading to filamentous fungi, the independent development of multicellularity led to organisms with a very different cellular organization compared with animals. Extant filamentous fungi are largely composed of syncytial colonies of “cells” which share a common cell wall based on chitin and beta-glucan polymers. In *Neurospora* we found evidence that GRHL plays a role in conidial cell wall formation and remodeling, in part through the regulation of chitinase 1 and various beta-glucan synthases. We also found that GRHL regulates genes involved in defense and virulence in the aerial hyphae and conidia of *Neurospora*. These effects were not so significant in the mycelia, which could be due to the fact that conidia are critical for asexual reproduction and are more likely to encounter novel and dangerous environments than mycelia.

Based on previously published GRH studies and the phenotype of the *Neurospora grhl/csp-2* mutant, as well as a comparison of the transcriptome profiles of a *Neurospora grhl* knockout and a *Drosophila grh* mutant, we present a model for the evolution of GRHL/CP2-family transcription factor function in the opisthokont lineage (Figure 6). We propose that the function of GRHL/CP2 proteins in the single-celled opisthokont last common ancestor was to regulate genes that contributed to both the formation and remodeling of an extracellular physical barrier (e.g., structural-biopolymer modifying enzymes and cell wall-associated proteins), and that it may also have regulated some genes that contributed to a defense-virulence “barrier”.

Strong evidence has accumulated that animal GRH-like proteins have a conserved function in the regulation of physical extracellular-barrier formation and wound healing in a wide variety of animal epithelial and epidermal tissues. For example, *Drosophila* GRH regulates the levels of genes encoding enzymes involved in cuticle melanization and chitin metabolism, cell adhesion proteins, and protein components of the cuticle. In mice, Grhl3 regulates the levels of genes that encode structural-barrier proteins in keratinocytes and the enzymes that crosslink such proteins, as well as cell-adhesion proteins and proteins that modulate the lipid composition of the epidermis [1], [3], [6], [13]–[15], [19]–[21], [24], [72], [73]. We propose that the original functions of Grainy head-like proteins in the opisthokont last common ancestor predisposed GRH-like proteins to regulate many aspects of extracellular-barrier formation and wound healing in early animals, as well as to evolve the related ability of regulating cell-cell adhesion genes in many epithelial tissues.

In the metazoan lineage, many types of epidermal barriers have evolved over time, including epithelia with chitin-based extracellular barriers (e.g., the arthropod epidermis), and it is interesting that chitin is one of the few extracellular structural biopolymers common to both fungi and animals. While chitin synthase itself does not appear to be regulated by GRH-like proteins in any system yet studied, it appears that GRH and GRH-like proteins of the CP2 superfamily regulate the expression of many genes involved in the formation and remodeling of chitin-based barriers, at least in *Neurospora* and *Drosophila*. It is also intriguing that *chitinase 1* in *Neurospora* and *chitinase 3* in *Drosophila* both appear to be strongly regulated by *GRHL* and GRH, respectively, consistent with an ancestral transcriptional control of chitinase expression by GRH-like proteins in the opisthokont last common ancestor. We believe it is possible that components of the ancestral opisthokont cell wall were repurposed (or redeployed) during the evolution of chitin-based apical extracellular barriers in some basal multicellular animals (Figure 6), with GRH proteins maintaining a role in barrier formation and remodeling during the process. A similar process may have occurred during the evolution of multicellular volvocine algae, as it has been proposed that the outer (tripartite) cell wall of unicellular algae evolved to become part of the apical extracellular barrier of multicellular algae [74]. This would have been independent of control by CP2 superfamily proteins, as sequenced genomes in the algal lineage do not encode recognizable members of this superfamily [28].

The evolution of multicellularity in fungi was presumably less complicated than in metazoans, as one can invoke incomplete cell division creating syncytial colonies of fungi. In this evolutionary scenario, the conservation of ancestral GRHL function with respect to barrier formation and remodeling would be straightforward, as the cell walls of the unicellular opisthokont last common ancestor and extant multicellular fungi would be very similar in structure and function. In addition to the greatly lowered expression of the *chitinase 1* gene (which is likely to be partially responsible for the conidial separation phenotype observed in *grhl* strains), we also found evidence that *Neurospora* GRHL plays a role in the expression of enzymes involved in the synthesis and remodeling of another key biopolymer of the fungal cell wall – beta-1,3-glucan. *GRHL* may turn out to have a more general role in promoting cell wall development, although we were unable to uncover phenotypic evidence for this, despite testing the growth of *grhl* mutant strains under several conditions shown elsewhere to inhibit the growth of *S. cerevisiae* strains with compromised cell walls (e.g., high-osmolarity media, high-temperature incubation, and media containing the chitin-binding molecule Calcofluor-White) [75] (data not shown). However, it is important to note that most cell wall integrity assays in *Neurospora* are based on mycelial cell wall growth, and if the *grhl* phenotypes manifest mainly in their non-dispersing conidia, the assays we used would probably not uncover them.

Dispersing conidia are a cell type very likely to encounter novel and dangerous environments, and one could imagine that a fast growing organism such as *Neurospora* would devote more resources towards protecting their spores than their mycelia. With this in mind, it was very interesting to see that many of the down-regulated genes with known or predicted functions on the *Neurospora grhl* AHC microarrays were classified as defense and virulence genes, and that many of the proteins encoded by these genes are predicted to be secreted. Extracellular barriers (such as the fungal cell wall or animal epidermis) act as passive defense mechanisms against infection, but they can also contain molecules that are actively hostile to pathogens [44], [45]. Furthermore, the distinction between defense and virulence in pathogenic fungi can be semantic – one way to become more virulent is to better defend yourself against your host, and vice versa. The deposition of defense-virulence factors into the fungal cell wall could be analogous to how many epithelial barriers throughout the animal and plant kingdoms produce antimicrobial peptides, both proactively and in response to infection (e.g., the *Drosophila* trachea and epidermis, mammalian lung and skin, and plant cuticles) [44], [45]. Unfortunately, *Neurospora crassa* does not have any characterized host-pathogen interactions, so we were unable to directly test the function of any of these genes in terms of their effects on virulence or defense. Experimental testing of the potential for GRHL proteins playing a direct role in defense and/or virulence will have to await studies in other ascomycete species with gene-knockout technology and well-characterized host-pathogen interactions.

While regulation of antimicrobial defense does not appear to be a major function of *Drosophila* GRH (at least in embryos), we did find a few innate immune genes that were significantly down-regulated on the *Drosophila grh^IM^* microarrays. We also found that knocking down GRH function in adult *Drosophila* increased susceptibility to septic (bacterial) wounding, without other discernable effects on overall health. Therefore, it is possible that GRH proteins might mediate some aspects of epidermal antimicrobial defense in *Drosophila*. There is as yet no functional evidence suggesting a role for mammalian GRH-family genes in epithelial antimicrobial defense, although the embryonic skin of mouse *Grhl3* mutants shows greatly reduced expression of one of the antimicrobial defensin genes, *Defa15* [21].

Although CP2 superfamily transcription factors with GRH-like properties were apparently encoded by the genome of the opisthokont last common ancestor, CP2/GRH-like proteins have been lost in many fungal lineages and, so far, have only been found in the genomes of a subset of the Ascomycota and Zygomycota [28]. On the face of it, this seems at odds with our proposal that GRH-like proteins are crucially linked to the regulation of extracellular-barrier formation, since many fungi with perfectly functional extracellular barriers (e.g., the well-studied ascomycete *Saccharomyces cerevisiae*, and basidiomycete mushrooms) lack any detectable genes of the CP2 or GRHL types. This discrepancy could be explained by the fact that, in Fungi, transcriptional batteries of genes that produce identical biological outputs can evolve to be regulated by different combinations of upstream transcription factors. For example, mating type in most ascomycete yeasts is regulated by the a2 transcription factor; however, this protein was lost in the lineage leading to *Saccharomyces cerevisiae*, which evolved a different combination of transcription factor inputs to determine mating type [76]. However, it is equally true that many animal transcription factor families, for hundreds of millions of years, have been regulating very similar developmental patterning or cell-type-specific properties during development [77] – a striking example of which is the conservation of GRH family function with respect to epithelial barrier formation in animals. It may be that the functions of animal transcription factors are somewhat more evolutionarily constrained than those of Fungi (perhaps due to differences in generation time, population size, or morphological complexity), and that Fungi are more likely to evolve new combinations of transcription factors to regulate core biological functions.

## Materials and Methods

### *Neurospora* Stocks, *grhl* Knockouts, and *grhl*/*csp-2* Complementation Assays

Wild-type strains [FGSC2489 (74-OR23-1V, mat A) and FGSC4200 (ORS-SL6, mat a)], *grhl* knockout strains [FGSC13563 (ΔNCU06095, mat A) and FGSC13564 (ΔNCU06095, mat a)], and the NHEJ-deficient strain [FGSC9720 (Δ*mus-52::bar*+; *his-3*, mat A)] were obtained from the Fungal Genetics Stock Center (FGSC) [78]. Stocks were maintained on minimal Vogel’s agar slants with 1.5% sucrose and appropriate supplements [36]. Genomic DNA for PCR analysis was obtained according to [79].

The isolation of the independently derived *grhl* deletion strains was performed by transforming a NCU06095-targeted hygromycin replacement cassette (courtesy of the Dunlap lab, Dartmouth) into FGSC9720, as described elsewhere [80]. Hygromycin-resistant colonies were selected, and homokaryonic *grhl* knockout strains were tested using PCR to verify loss of the *grhl* locus. All strains missing the *grhl* locus displayed the conidial separation phenotype. These new strains (Δ*mus-52::bar+*; Δ*grhl::hyg+*; *his-3*, mat A) were also used in the complementation-assay fusings described below. The primer sequences for verifying the *grhl* knockouts and for detecting *grhl* transcripts (Figures 2A and B) were as follows: grh-For – CACCAGTCAAGCTGGCATC –and grh-Rev – GGCTTATGTCGCTGCTTTTC. Positive control primers were as follows: actin-For – ATCCGACACTTTTCGTCACC – and actin-Rev – TGCAACAACCACCTCTCAAG.

Genetic complementation assays between *grhl* and *csp-2* were carried out by fusing one of the independently derived *grhl* deletion strains described above (Δ*mus-52::bar+*; Δ*grhl::hyg+*; *his-3*, mat A) to ten different isolates of *csp-2*; *bd*; *inos*. The *csp-2*; *bd*; *inos* strains were created using standard crossing methods (*csp-2; bd* × *inos*). Exactly as expected, only half (five) of the fusings were viable on minimal media (due to opposite mating-type incompatibility), all of which displayed the conidial separation phenotype. Using PCR, the *csp-2*/*grhl* heterokaryons were verified as positive for the *grhl* locus, which is consistent with the deletion/stop codon in *csp-2* strains (data not shown).

### *Neurospora* GRHL and *Drosophila* GRH Protein Production and Gel-shifts

The full-length *Drosophila grh* coding sequence was cloned into the plasmid pcDNA 3.1/myc-His(-) A (Invitrogen) as described elsewhere [4]. The full-length *Neurospora grhl* coding sequence was amplified using the Phusion polymerase (New England Biolabs) from an oligo-dT-primed cDNA library (RETROscript kit, Ambion). The primers Grhl5’XbaIKozak –GCGTCTAGAGCCACCATGTTCAGTCAACGAACAAG – and Grhl3’HindIII –CGCAAGCTTGTAGAGCAGTCGCAGTTCAT – were used to introduce a Kozak sequence (for efficient translation) and restriction endonuclease sites. The fragment was cloned into pcDNA 3.1/myc-His(-) A using the *XbaI* and *HindIII* sites in the multiple cloning site. The insert was fully sequenced and was found to be identical in sequence and exon structure to that predicted by the Broad Institute *Neurospora* database.

GRH and GRHL proteins were translated using the TNT T7 Quick Coupled Transcription/Translation System (Promega) by adding 1 µg of template to each master mix aliquot, according to the manufacturer’s instructions. Protein expression levels were assayed by Western blotting, using antibodies against the C-terminal Myc tags, as described elsewhere [4]. The translated proteins were directly used in the gel-shift assays, as freezing was found to negatively affect DNA-binding activity. For each oligonucleotide pair, 500 pmol of each were annealed in a final volume of 100 µl in annealing buffer (10 mM Tris-HCl pH 7.5, 20 mM NaCl) by heating to 95°C for 5 min and slowly cooling to 25°C. Then, 5 pmol of double-stranded oligonucleotides was labeled with polynucleotide kinase (New England BioLabs) in the presence of ATP-[^32^P] for 30 min at 37°C. The double-stranded probes were purified using the QIAquick Nucleotide Removal Kit (Qiagen). Next, 10–20 fmol of radiolabeled double-stranded oligonucleotides and 1.5 µl of protein from the in vitro transcription/translation reactions were added to 10 µl binding buffer [25 mM Hepes pH 7.9, 100 mM KCl, 1 mM DTT, 1% polyvinylalcohol, 1% Nonidet P-40, 0.1% BSA, 10% glycerol, and 20 µg/ml poly(dI-dC)] and incubated with DNA for 30 min at 4°C. The binding reaction was then electrophoresed through a 4% native polyacrylamide gel in 0.5× TBE at 4°C. Gels were dried and autoradiographed with the use of intensifying screens.

### *Neurospora* and *Drosophila* Microarray Sample Collection

*Neurospora* samples for microarray analysis were collected according to the following procedures. Seeder slants of wild-type (FGSC2489) and *grhl* (FGSC13563) strains were grown for 3 days at 30°C under a 12 h light/dark cycle, and conidia were harvested in 1 ml H_2_0. As the *csp* phenotype makes homogenous resuspension of *grhl* conidia impossible, accurate conidial counts of the suspensions could not be obtained. Therefore, plates and flasks were innoculated with approximately equivalent masses of conidia suspended in water. As *Neurospora* comes to confluence very quickly on plates, and growth in liquid culture for short periods of time should not be nutrient limiting, we believe the number of starting conidia was not crucial to these experiments. The following collection procedures were carried out in triplicate for both wild-type and *grhl* samples. 1) “ALL” samples were collected by densely plating conidia on minimal Vogel’s agar medium +1.5% sucrose in 10 cm Petri dishes that had been overlain with disks of cellophane (VWR, 100357–652). After 48 h at 30°C under a 12 h light/dark cycle, the plates were densely covered with conidiating colonies. Samples were scraped off the cellophane using cell-scrapers, submerged in 5 ml Trizol (Invitrogen), and quickly frozen in liquid nitrogen. 2) “AHC” samples were collected by densely plating conidia on minimal Vogel’s agar medium +1.5% sucrose in a deep 10 cm Petri dishes. Disks of medium gauge wire mesh were suspended ∼0.5 cm above the surface of the agar using a ring of plastic tubing around the periphery of each Petri dish. After 48 h at 30°C under a 12 h light/dark cycle, the aerial hyphae and conidia had grown abundantly through the mesh. The mesh disks were carefully peeled off, and the adherent cells were harvested in H_2_0 using cell-scrapers. Samples were dried by vacuum filtration, removed from the filter paper using cell-scrapers, submerged in 5 ml Trizol, and quickly frozen in liquid nitrogen. 3) “MYC” samples were collected by inoculating 25 ml of liquid Vogel’s medium +1.5% sucrose in 125 ml Erlenmeyer flasks with sponge stoppers. After 28 h at 28°C with constant shaking in the dark, log-phase mycelial mats were recovered by vacuum filtration, removed from the filter paper using cell-scrapers, and frozen in 2 ml Trizol with liquid nitrogen.

To obtain total RNA from the *Neurospora* samples for microarray analysis, we followed a protocol similar to that reported elsewhere [81]. Samples in Trizol were thawed and quickly homogenized by vortexing and passing through a P1000 pipet tip multiple times to break up large clumps. Approximately 100 µl of cells were placed in an eppendorf tube with 1 ml Trizol and 200 mg of 0.5 mm Zirconia/Silica Beads (Biospec). Samples were disrupted twice with a MiniBeadBeater (Biospec) at maximum speed for 30 s each time. RNA was then extracted using standard Trizol procedures and resuspended in 100 µl H_2_0. RNA was quantified in 10 mM Tris pH 7.5, and 50 µg of total RNA was cleaned further using the RNeasy miniprep kit (Qiagen). RNA integrity was assayed by gel electrophoresis, RT-PCR against several developmentally dynamic genes [81], and Bioanalyzer (Agilent) analysis (data not shown).

The following *Drosophila* embryo collection procedures were carried out in duplicate. To aid in the collection of homozygous *grh*-deficient embryos, the *cn, grh^IM^, bw, sp* chromosome [2] was placed over the fluorescent balancer *CyO, Kruppel-GFP* (*CKG*) [82]. Heterozygous collections of embryos (+; *cn, grh^IM^, bw, sp*/*CKG*; +) were allowed to develop at 25°C until ∼15–18 h of age. Embryos were aligned on a thin agar slab on a slide, and GFP-negative embryos (*grh^IM^* homozygotes) were selected using epifluorescent microscopy. Gut autofluorescence in the GFP channel allowed for the selection of viable and properly aged embryos (late stage 16 and early stage 17 [60]) using gut morphology as a guide. Correspondingly staged wild-type (*y*; *cn, bw, sp*; *+*) embryos were similarly raised and selected using gut autofluorescence as a guide. Approximately 500 mutant and wild-type embryos were collected and stored frozen in Trizol. Embryos were ground in Trizol using a pestle, RNA was purified using standard Trizol procedures, and RNA integrity was assayed as described above.

### Microarray Design and Analysis

*Neurospora* microarrays were custom synthesized by Agilent using the sequences from the *Neurospora crassa* arrays available from the FGSC [81], [83]. All probe sequences were shortened from 70 mers to 60 mers by removing the first ten 5′ nucleotides. A total of 10,526 unique spots were printed on each chip, corresponding to predicted genes from several databases. Once a finalized list of significant genes was obtained, probe sequences were BLASTed against the *Neurospora crassa* genome to verify the Broad (or MIPS) gene ID annotations.

Predesigned *Drosophila melanogaster* arrays were ordered from Agilent (Design ID # 18972). A total of 43,603 spots were printed on each chip, which mapped to ∼13,000 unique FlyBase genes. Fluorescence values from redundant probes (or unique probes targeting the same gene) were grouped, and only the highest fold-change values were used in these analyses. Once a finalized list of significant genes was obtained, probe sequences were BLASTed against the *Drosophila melanogaster* genome to verify the FlyBase CG # annotations.

RNA labeling, hybridizations, fluorescent quantification, data normalization, FDR calculations [84], and GO annotations (Tables 1 and S1) were carried out by the Biogem Core facility (UC San Diego); see Text S1 for an in-depth description of the microarray analyses. Manual *Neurospora* gene classifications (Figure 3) were carried out by consulting the Broad Institute *Neurospora crassa* database (www.broadinstitute.org/annotation/genome/neurospora/MultiHome.html), the MIPS *Neurospora crassa* database (mips.helmholtz-muenchen.de/genre/proj/ncrassa), and the Functional Catalog (FunCat) [41] classifications found in the MIPS database (mips.helmholtz-muenchen.de/genre/proj/ncrassa/Search/Catalogs/searchCatfirstFun.html), as well as with literature and homology searches. Both the SignalP (www.cbs.dtu.dk/services/SignalP) and TargetP (www.cbs.dtu.dk/services/TargetP) servers were used to look for secretion signals in the down-regulated *Neurospora* proteins. Manual *Drosophila* gene classifications (Table 2) were carried out by consulting Flybase (flybase.org) and The Gene Ontology (www.geneontology.org), as well as with literature and homology searches. The NCBI BLAST (blast.ncbi.nlm.nih.gov/Blast.cgi) and JGI (genome.jgi.doe.gov/genome-projects) search tools were used extensively in these analyses. The normalized microarray results have been deposited in the NCBI Gene Expression Omnibus (www.ncbi.nlm.nih.gov/geo), and the accession numbers for the *Neurospora* and *Drosophila* datasets are GSE35017 and GSE34997, respectively.

### *Drosophila* RNAi Strains and Conditions

Flies were raised on the standard *Drosophila* medium at 25 or 29°C. The *hs-GAL4* driver was located on the third chromosome, and the flies were obtained from the Bloomington Stock Center. The *e13C-GAL4* [70] flies were a gift from Dr. Norbert Perrimon at Harvard University. The *UAS-GRH^RNAi^* flies were obtained from the Vienna Drosophila RNAi Center (http://stockcenter.vdrc.at/control/main).

### Epidermal Tissue Preparation and Immunostaining

Wandering third-instar larvae were rinsed in phosphate-buffered saline (PBS), dissected in Brower Fixative (0.15 M PIPES pH 6.9, 3 mM MgSO_4_, 1.5 mM EGTA, and 1.5% NP40) with 4% formaldehyde (ultrapure, methanol-free from Polysciences Inc.), and fixed for 2 h at 4°C. To visualize melantoic spots, fixed larval tissues were washed in PBS with 0.1% Tween 20 and 0.1% Triton X-100 (PBTwx), mounted in Prolong Gold anti-fade reagent (Invitrogen), and imaged using phase-contrast microscopy on a Leica DM 2500 microscope. Epidermal tissues from adult abdomens were also dissected in Brower Fixative with 4% formaldehyde, fixed for 2 h at 4°C, and excess abdominal fat was removed by gentle aspiration.

For immunostaining, fixed epidermal tissues were washed in PBTwx, then incubated in a blocking solution of PBTwx with Western blocking reagent (WBR; Roche) for 1 h at room temperature. Incubations with primary antibodies were performed in PBT + WBR at 4°C overnight, and incubations with fluorescently labeled secondary antibodies were performed in PBT + WBR at room temperature for 2 hours. Primary antibodies utilized in this paper were as follows: guinea pig anti-GRH at a 1∶300 dilution [6] and mouse anti-Fasciclin 3 (7G10 concentrate, from the Developmental Studies Hybridoma Bank) at a 1∶400 dilution. Fluorescently labeled secondary antibodies from Invitrogen (Alexa Fluor 555 goat anti-guinea pig IgG and Alexa Fluor 488 donkey anti-mouse IgG) were used at 1∶400 dilutions. Tissues were mounted in Prolong Gold.

All fluorescent images were collected using a Leica SP2 laser-scanning confocal microscope, with identical instrument settings (at non-saturated gain levels) for both experimental and control samples. Optical sections were scanned at 0.6–0.8 µm thicknesses, and maximum-projection images are shown in Figures 4 and 5.

### Drying Assays for Wandering Third-instar *Drosophila* Larvae

To cause clean injury, larvae were first rinsed in PBS, placed in a small drop of PBS on a black rubber block, and were punctured with a sterile 0.125 mm tungsten needle (Fine Science Tools) through their posterior-lateral epidermis, as described elsewhere [85], [86]. After epidermal injury, larvae were placed into either a Petri dish (60×15 mm) containing Whatman 3 MM chromatography paper moistened with PBS (moist conditions), or an empty Petri dish (dry conditions), and raised at 25°C. Mock-treated larvae were treated as above, except without any epidermal injury. Individual drying assays were performed using at least 40 larvae for each genotype, and each assay was repeated at least three times. Images of wandering third-instar larvae were obtained using a SteREO Discovery.V12 stereomicroscope (Zeiss), and images of representative larvae are presented in Figure 4.

### Clean and Septic Epidermal Injury Experiments in Adult *Drosophila*

Approximately 24 h old male flies were heat shocked for 3 h at 37°C once a day for four consecutive days; flies were raised at 29°C between and after heat shock treatments. Clean or septic injuries were performed on the fifth day after eclosion using a 0.25 mm tungsten needle to puncture their dorsal abdomens, as described elsewhere [85], [86]. For septic injury, needles were dipped in gram-negative *Erwinnia carotovora carotovora 15* (*Ecc15*) or gram-positive *Microccocus luteus* (*M. luteus*) bacterial solutions prior to injury. Mock-treated control flies were heat shocked and raised as described above, except without epidermal injury. Individual survival assays were carried out with at least 50 adult male flies for each genotype, and each assay was repeated at least three times. Injured males were placed into clean vials to monitor survival, with eight to ten flies per vial to avoid crowding. Surviving adult flies were counted and transferred to fresh vials every two days for ten total days. The averages of three experimental replicates are shown with the standard errors of the mean.

## Supporting Information

Figure S1 **The mutation responsible for the *csp-2^FS590^* allele is in the *grhl* gene (NCU06095).** (**A**) The wild-type amino acid sequence of GRHL/Csp-2. The serine whose codon is mutated in the *csp-2^FS590^* allele is highlighted in blue. The generation and initial characterization of this UV-induced mutation is described in Selitrennikoff et al. (1974). The residues of the DNA-binding domain are shown in bold. (**B**) Codon S509 contains a one bp deletion (-) in the *csp-2^FS590^* allele, which results in a frame-shift mutation leading to a premature stop codon (*) after 14 out-of-frame codons. This mutant allele is predicted to encode a truncated version of the GRHL/Csp-2 protein lacking the proper 285 C-terminal amino acids. We also identified a C>A mutation in codon 510, although whether this mutation existed in the parental strain prior to mutagenesis is unclear.
(TIF)

Figure S2 **Quantitative RT-PCR verification of the fold changes observed on the** ***Neurospora grhl*** **AHC microarrays.** Quantitative RT-PCR (qPCR) was carried out on a selection of ten genes (five up- and five down-regulated) seen to be misregulated on the *Neurospora grhl* AHC microarrays. Genes were chosen to span a wide range of fold changes. The qPCR results verify the directionality of the fold changes seen on the microarrays, as well as (in most cases) the approximate fold-change values. Results were analyzed using two different housekeeping genes as controls – *actin* and *cbp*. Labels correspond to the following genes: NCU04883– *chitinase 1*; NCU04850– *exo-beta-1,3-glucanase*; NCU07787– *cerato-platanin*; NCU10051– *flavohemoglobin*; NCU03415– *aldehyde dehydrogenase*; NCU07821– *dimethylaniline monooxygenase*; NCU04533– *abundant perithecial protein*; NCU07610– *taurine dioxygenase*; NCU07819– *alpha-ketoglutarate-dependent taurine dioxygenase*; and NCU07232– *heat shock protein 30*. Primer sequences were as follows: NCU04883– TACCTCTGCTGACACCAACG and CTTTGAGGTTGGCAAAGGAG; NCU04850– TCTCTACAGCGGTCGTGGTC and CCGACCATGATATCGACGAC; NCU07787– AAGATCCTCAGCCTTTTCACC and GTCGTAGCCCGTGTCGTAG; NCU10051– ATCTGCATTTGGCGGATAAG and CCGTAGCAAAAAGCTCCAAG; NCU03415– CTTAGGGCTGGTACCGTCTG and ACCGATACCGGACTCCTTG; NCU07821– TACCCGGGTCTGTTGTTCTC and GGGAGAAAGGGGTAGGACAC; NCU04533– CTTGAAGGTGGATGCGAGAG and GACCAGCCCATACTCGTCTC; NCU07610– GATTTGCAGGTGCGGTTTAG and ATCCAACCGTACGATTACCG; NCU07819– AAAGCATTGTGGGTGAATCG and TCAGAATCACATCGCTCTCG; and NCU07232– AGCGCAGCTATGGAGAGTTC and TATCCTGATCCACCGGAGTC.
(TIF)

Figure S3 **Up-regulated genes from the** ***Neurospora grhl*** **Aerial Hyphae and Conidia microarray samples.** A manual classification of the significantly up-regulated genes from the *Neurospora grhl* AHC microarrays. **“Broad ID”** entries correspond to the gene IDs found in the Broad Institute *Neurospora crassa* database. Italicized entries in this column refer to probes that do not correspond to genes in the Broad database, but which correspond to genes in the MIPS database. **“Gene name or Description”** entries were based on the annotations found in the Broad and MIPS databases, as well literature and homology searches. **“Fold (wt value)”** entries indicate the fold changes observed in *grhl* mutant aerial hyphae and conidia relative to wild type; wild-type microarray fluorescence values are shown in parentheses (the background level was ∼100 units). **“FDR”** entries indicate the False Discovery Rate values calculated for each gene; only genes with FDR values less than 0.01 are shown. **Columns 1–9** of the grid represent a simplification of the FunCat classification system; solid-colored blocks indicate those genes are classified in the corresponding FunCat categories; dashes indicate that we found evidence in the literature to suggest these genes belong in the corresponding categories. **Column 10** of the grid indicates whether the encoded proteins are predicted to be secreted, according to the or TargetP (T) prediction algorithm.
(TIF)

Figure S4 **The lesion responsible for the** ***grh^IM^*** **allele is a stop-codon introduction shortly into the DNA-binding domain.** (**A**) RT-PCR demonstrates that *grh^IM^* embryos still produce *grh* transcripts at roughly the same levels as wild-type embryos; RT-PCRs were carried out with biological replicates. (**B**) A schematic showing the location of the TAT>TAA stop-codon introduction in the *grh^IM^* mRNA, shortly after the start of the DNA-binding domain (tyrosine Y29, from the “*D.mel* GRH” protein sequence in Figure 1B). (**C–F**) Sequencing reactions from both RNA and genomic DNA templates unambiguously verify this mutation: homozygous deficiency (*cn, grh^IM^, bw, sp*) RNA from embryos (C), wild-type (*y*; *cn, bw, sp*) RNA from embryos (D), heterozygous (*cn, grh^IM^, bw, sp/CyO, Kruppel-GFP*) genomic DNA from adults (E), and wild-type (*w^1118^*) DNA from adults (F).
(TIF)

Figure S5 **Quantitative RT-PCR verification of the fold changes observed on the** ***Drosophila grh^IM^*** **embryo microarrays.** Quantitative RT-PCR (qPCR) was carried out on a selection of eight genes (three up- and five down-regulated) seen to be misregulated on the *Drosophila grh^IM^* microarrays. Genes were chosen to span a wide range of fold changes. The qPCR results verify the directionality of the fold changes seen on the microarrays, as well as (in most cases) the approximate fold-change values. Results were analyzed using the housekeeping gene *Rp49* (CG7939) as a control. Primer sequences were as follows: *Lcp4*– TTCAAGATCCTGCTTGTCTGC and GACATCGTTGACCAGCTCCT; *Chitinase 3*– TACGTCGAGCGAAGCTGTC and CTGGTTTGATCCCAATGAGG; *Cpr67Fa1*– GCCAGCAAAGATGTTCCG and ATGTAGGCACCAGCTTCCTG; *TepII* – GAATCATGAACTGATCCCGAAG and TCCGTCTTGTCAGCCTCTTC; *eater* – GGATGGCCATGAAAAGAGTG and CCACGTGATATGAGCGTTTC; *Catalase* – TGCTGAGGTGGAGCAGATC and AGGAGAACAGACGACCATGC; *Cyp6w1*– GAAGATTGGAAAGAACTTGCAG and CGGGAGCATAGATCCTTCAC; and *drosomycin 5*– GCCGACTGTCTCTCTGGAAG and CAGGTCTCGTTGTCCCAGAC.
(TIF)

Table S1 **Significantly enriched Gene Ontology categories for the misregulated genes on the** ***Drosophila grh^IM^*** **embryo microarrays.** The enriched Gene Ontology (GO) “Molecular Function”, “Biological Process”, and “Cellular Component” categories for all misregulated genes from the *Drosophila grh^IM^* embryo microarrays.
(DOC)

Text S1 **Statistical and Bioinformatical Analyses of Microarray Data.**
(DOC)
