# Supplementary figures and images for: The Functions of Grainy Head-Like Proteins in Animals and Fungi and the Evolution of Apical Extracellular Barriers
Source: PLoS One. 2012 May 9;7(5):e36254. doi: 10.1371/journal.pone.0036254 (PMC3348937; doi:10.1371/journal.pone.0036254)

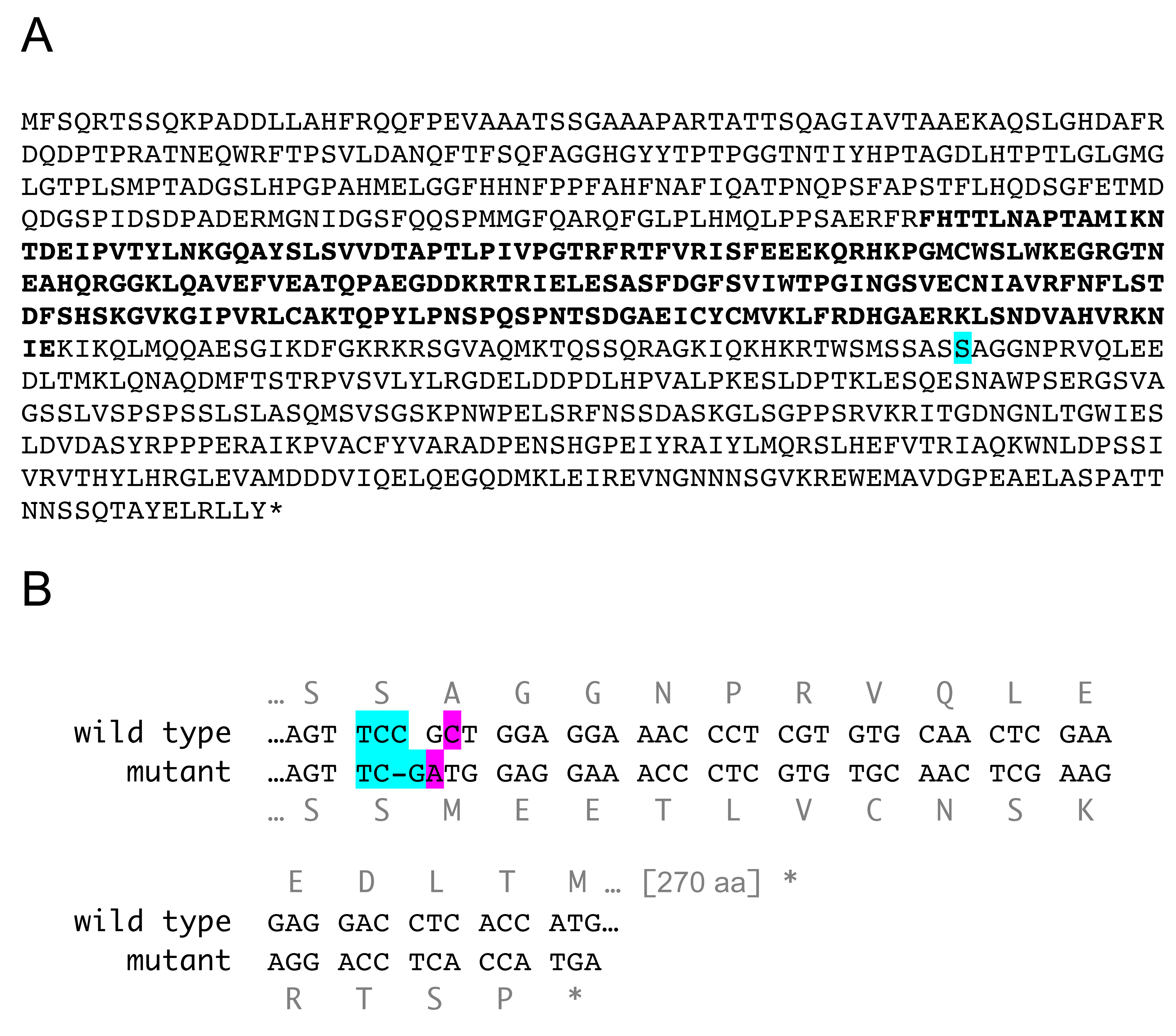

Supplement: Figure S1 — The mutation responsible for the csp-2FS590 allele is in the grhl gene (NCU06095). (A) The wild-type amino acid sequence of GRHL/Csp-2. The serine whose codon is mutated in the csp-2FS590 allele is highlighted in blue. The generation and initial characterization of this UV-induced mutation is described in Selitrennikoff et al. (1974). The residues of the DNA-binding domain are shown in bold. (B) Codon S509 contains a one bp deletion (-) in the csp-2FS590 allele, which results in a frame-shift mutation leading to a premature stop codon (*) after 14 out-of-frame codons. This mutant allele is predicted to encode a truncated version of the GRHL/Csp-2 protein lacking the proper 285 C-terminal amino acids. We also identified a C>A mutation in codon 510, although whether this mutation existed in the parental strain prior to mutagenesis is unclear. (TIF) [file pone.0036254.s001.tif]

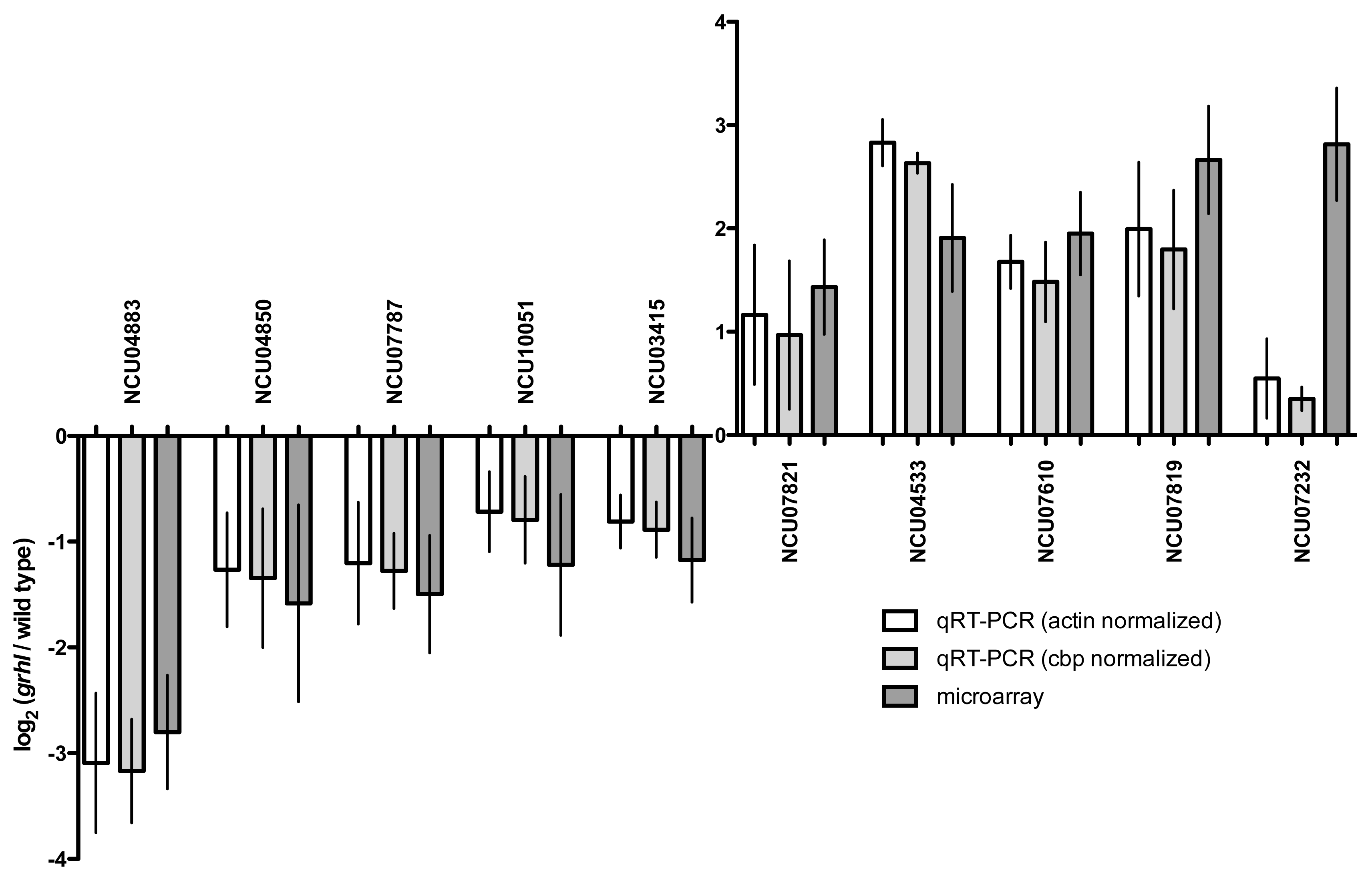

Supplement: Figure S2 — Quantitative RT-PCR verification of the fold changes observed on the Neurospora grhl AHC microarrays. Quantitative RT-PCR (qPCR) was carried out on a selection of ten genes (five up- and five down-regulated) seen to be misregulated on the Neurospora grhl AHC microarrays. Genes were chosen to span a wide range of fold changes. The qPCR results verify the directionality of the fold changes seen on the microarrays, as well as (in most cases) the approximate fold-change values. Results were analyzed using two different housekeeping genes as controls – actin and cbp. Labels correspond to the following genes: NCU04883– chitinase 1; NCU04850– exo-beta-1,3-glucanase; NCU07787– cerato-platanin; NCU10051– flavohemoglobin; NCU03415– aldehyde dehydrogenase; NCU07821– dimethylaniline monooxygenase; NCU04533– abundant perithecial protein; NCU07610– taurine dioxygenase; NCU07819– alpha-ketoglutarate-dependent taurine dioxygenase; and NCU07232– heat shock protein 30. Primer sequences were as follows: NCU04883– TACCTCTGCTGACACCAACG and CTTTGAGGTTGGCAAAGGAG; NCU04850– TCTCTACAGCGGTCGTGGTC and CCGACCATGATATCGACGAC; NCU07787– AAGATCCTCAGCCTTTTCACC and GTCGTAGCCCGTGTCGTAG; NCU10051– ATCTGCATTTGGCGGATAAG and CCGTAGCAAAAAGCTCCAAG; NCU03415– CTTAGGGCTGGTACCGTCTG and ACCGATACCGGACTCCTTG; NCU07821– TACCCGGGTCTGTTGTTCTC and GGGAGAAAGGGGTAGGACAC; NCU04533– CTTGAAGGTGGATGCGAGAG and GACCAGCCCATACTCGTCTC; NCU07610– GATTTGCAGGTGCGGTTTAG and ATCCAACCGTACGATTACCG; NCU07819– AAAGCATTGTGGGTGAATCG and TCAGAATCACATCGCTCTCG; and NCU07232– AGCGCAGCTATGGAGAGTTC and TATCCTGATCCACCGGAGTC. (TIF) [file pone.0036254.s002.tif]

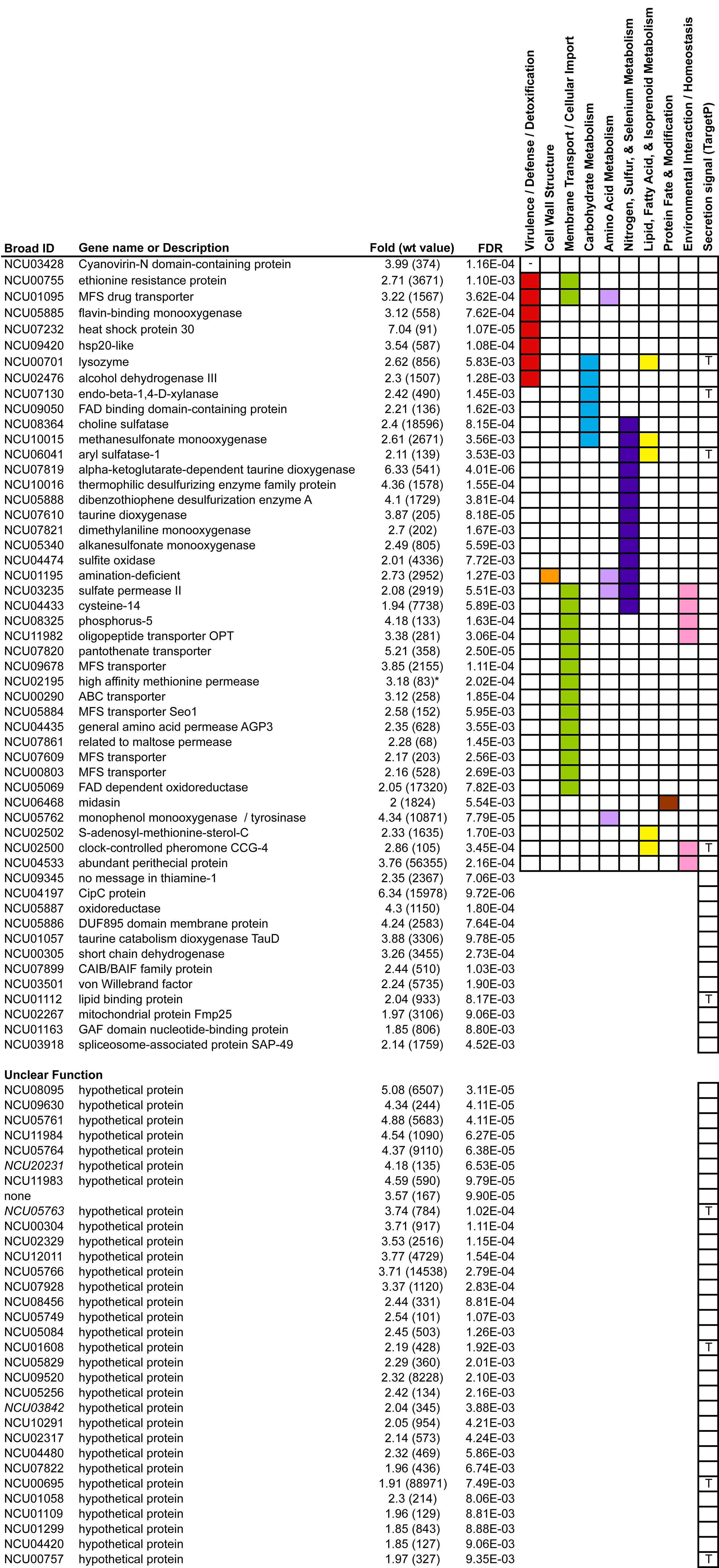

Supplement: Figure S3 — Up-regulated genes from the Neurospora grhl Aerial Hyphae and Conidia microarray samples. A manual classification of the significantly up-regulated genes from the Neurospora grhl AHC microarrays. “Broad ID” entries correspond to the gene IDs found in the Broad Institute Neurospora crassa database. Italicized entries in this column refer to probes that do not correspond to genes in the Broad database, but which correspond to genes in the MIPS database. “Gene name or Description” entries were based on the annotations found in the Broad and MIPS databases, as well literature and homology searches. “Fold (wt value)” entries indicate the fold changes observed in grhl mutant aerial hyphae and conidia relative to wild type; wild-type microarray fluorescence values are shown in parentheses (the background level was ∼100 units). “FDR” entries indicate the False Discovery Rate values calculated for each gene; only genes with FDR values less than 0.01 are shown. Columns 1–9 of the grid represent a simplification of the FunCat classification system; solid-colored blocks indicate those genes are classified in the corresponding FunCat categories; dashes indicate that we found evidence in the literature to suggest these genes belong in the corresponding categories. Column 10 of the grid indicates whether the encoded proteins are predicted to be secreted, according to the or TargetP (T) prediction algorithm. (TIF) [file pone.0036254.s003.tif]

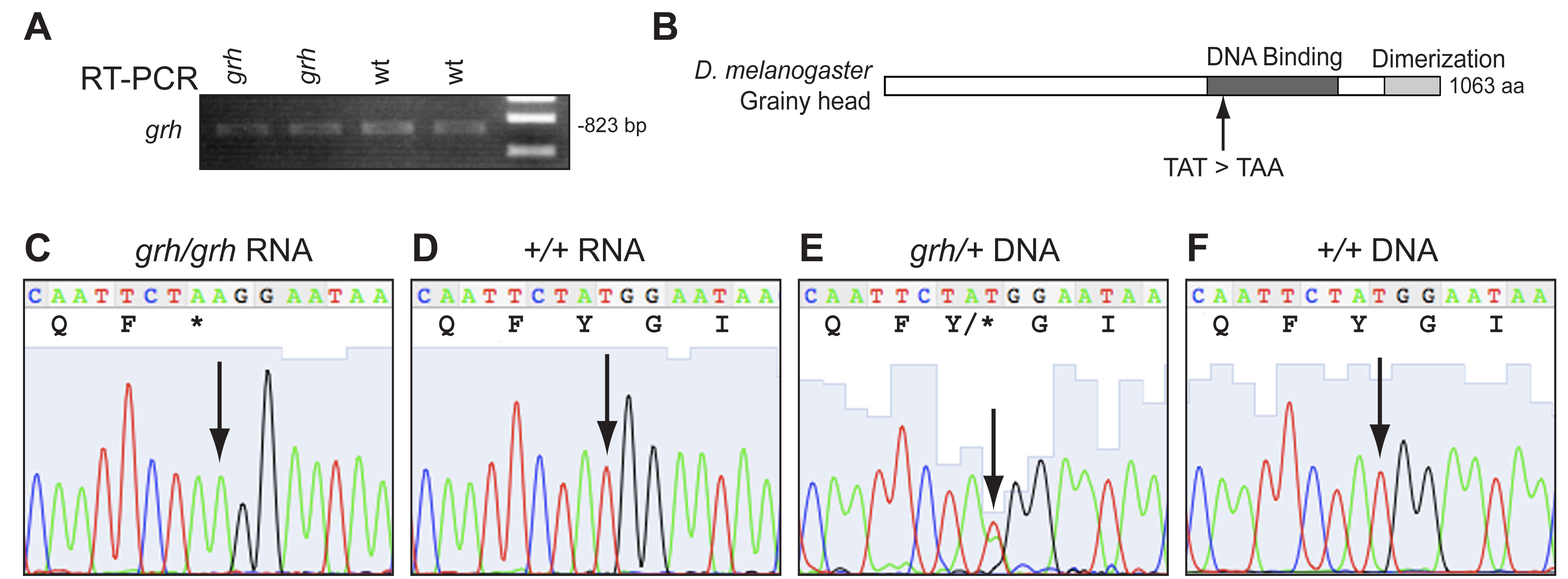

Supplement: Figure S4 — The lesion responsible for the grhIM allele is a stop-codon introduction shortly into the DNA-binding domain. (A) RT-PCR demonstrates that grhIM embryos still produce grh transcripts at roughly the same levels as wild-type embryos; RT-PCRs were carried out with biological replicates. (B) A schematic showing the location of the TAT>TAA stop-codon introduction in the grhIM mRNA, shortly after the start of the DNA-binding domain (tyrosine Y29, from the “D.mel GRH” protein sequence in Figure 1B). (C–F) Sequencing reactions from both RNA and genomic DNA templates unambiguously verify this mutation: homozygous deficiency (cn, grhIM, bw, sp) RNA from embryos (C), wild-type (y; cn, bw, sp) RNA from embryos (D), heterozygous (cn, grhIM, bw, sp/CyO, Kruppel-GFP) genomic DNA from adults (E), and wild-type (w1118) DNA from adults (F). (TIF) [file pone.0036254.s004.tif]

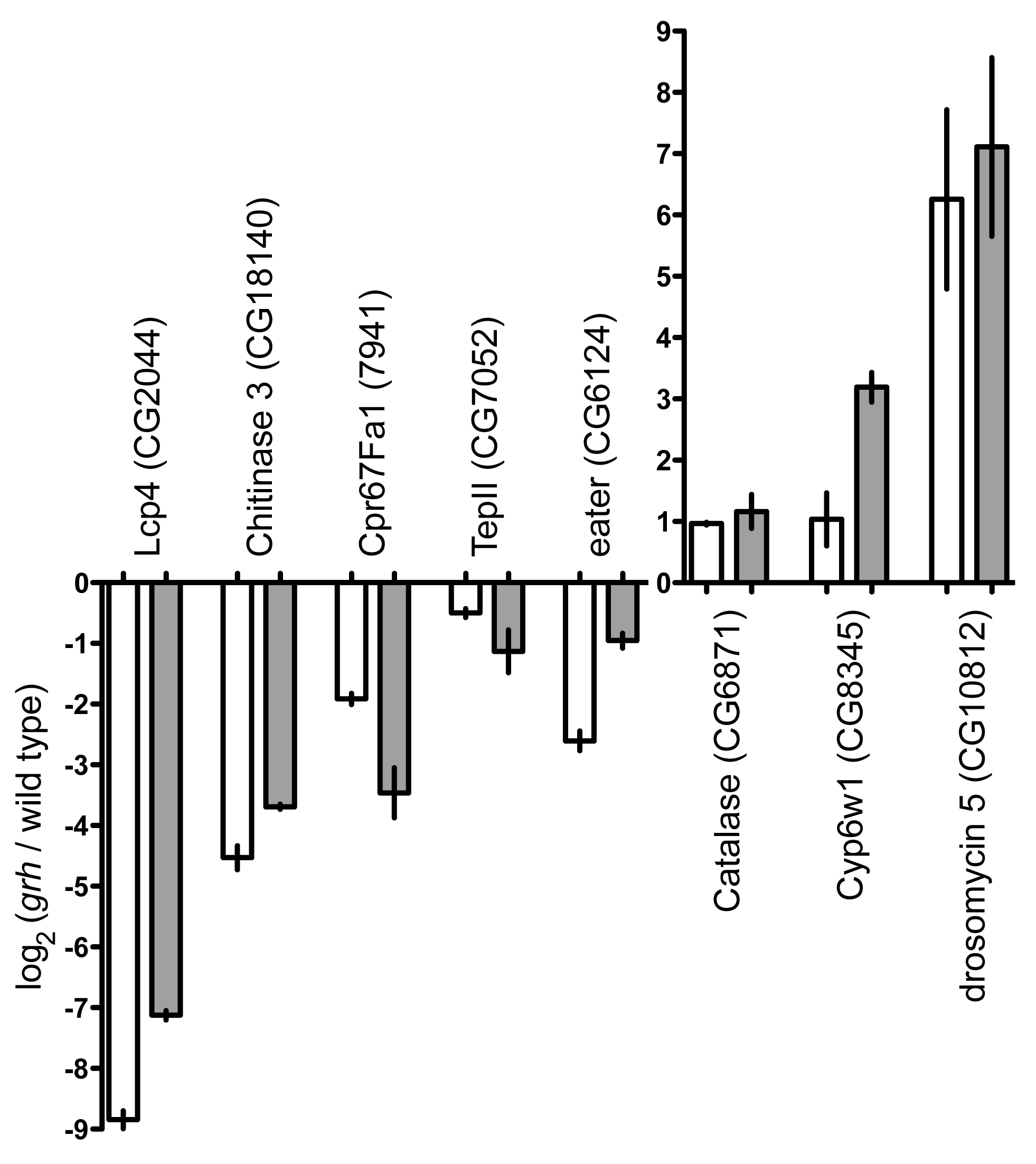

Supplement: Figure S5 — Quantitative RT-PCR verification of the fold changes observed on the Drosophila grhIM embryo microarrays. Quantitative RT-PCR (qPCR) was carried out on a selection of eight genes (three up- and five down-regulated) seen to be misregulated on the Drosophila grhIM microarrays. Genes were chosen to span a wide range of fold changes. The qPCR results verify the directionality of the fold changes seen on the microarrays, as well as (in most cases) the approximate fold-change values. Results were analyzed using the housekeeping gene Rp49 (CG7939) as a control. Primer sequences were as follows: Lcp4– TTCAAGATCCTGCTTGTCTGC and GACATCGTTGACCAGCTCCT; Chitinase 3– TACGTCGAGCGAAGCTGTC and CTGGTTTGATCCCAATGAGG; Cpr67Fa1– GCCAGCAAAGATGTTCCG and ATGTAGGCACCAGCTTCCTG; TepII – GAATCATGAACTGATCCCGAAG and TCCGTCTTGTCAGCCTCTTC; eater – GGATGGCCATGAAAAGAGTG and CCACGTGATATGAGCGTTTC; Catalase – TGCTGAGGTGGAGCAGATC and AGGAGAACAGACGACCATGC; Cyp6w1– GAAGATTGGAAAGAACTTGCAG and CGGGAGCATAGATCCTTCAC; and drosomycin 5– GCCGACTGTCTCTCTGGAAG and CAGGTCTCGTTGTCCCAGAC. (TIF) [file pone.0036254.s005.tif]
