# Supplementary material for: The Functions of Grainy Head-Like Proteins in Animals and Fungi and the Evolution of Apical Extracellular Barriers
Source: PLoS One. 2012 May 9;7(5):e36254. doi: 10.1371/journal.pone.0036254 (PMC3348937; doi:10.1371/journal.pone.0036254)
Supplement: Table S1 — Significantly enriched Gene Ontology categories for the misregulated genes on the Drosophila grhIM embryo microarrays. The enriched Gene Ontology (GO) “Molecular Function”, “Biological Process”, and “Cellular Component” categories for all misregulated genes from the Drosophila grhIM embryo microarrays. (DOC) [file pone.0036254.s006.doc]

| **GO ID** | **Molecular Function** | **p** | **Bonferroni p** |
| --- | --- | --- | --- |
| 42302 | structural constituent of cuticle | 9.66E-19 | 1.02E-15 |
| 5214 | structural constituent of chitin-based cuticle | 2.20E-17 | 2.32E-14 |
| 4252 | serine-type endopeptidase activity | 7.24E-17 | 7.63E-14 |
| 17171 | serine hydrolase activity | 1.46E-16 | 1.54E-13 |
| 8236 | serine-type peptidase activity | 1.75E-16 | 1.85E-13 |
| 70011 | peptidase activity, acting on L-amino acid peptides | 8.26E-12 | 8.71E-09 |
| 4175 | endopeptidase activity | 1.86E-11 | 1.96E-08 |
| 8010 | structural constituent of chitin-based larval cuticle | 4.56E-11 | 4.81E-08 |
| 8233 | peptidase activity | 1.11E-10 | 1.17E-07 |
| 8061 | chitin binding | 1.76E-10 | 1.85E-07 |
| 1871 | pattern binding | 4.51E-10 | 4.75E-07 |
| 30247 | polysaccharide binding | 4.51E-10 | 4.75E-07 |
| 16614 | oxidoreductase activity, acting on CH-OH group of donors | 2.64E-09 | 2.79E-06 |
| 30246 | carbohydrate binding | 7.23E-09 | 7.62E-06 |
| 3796 | lysozyme activity | 3.58E-08 | 3.77E-05 |
| 16616 | oxidoreductase activity, acting on the CH-OH group of donors, NAD or NADP as acceptor | 3.11E-07 | 3.28E-04 |
| 16491 | oxidoreductase activity | 4.20E-07 | 4.43E-04 |
| 4497 | monooxygenase activity | 4.22E-07 | 4.45E-04 |
| 5506 | iron ion binding | 4.30E-07 | 4.53E-04 |
| 15020 | glucuronosyltransferase activity | 5.34E-07 | 5.63E-04 |
| 4553 | hydrolase activity, hydrolyzing O-glycosyl compounds | 1.50E-06 | 1.58E-03 |
| 61135 | endopeptidase regulator activity | 2.66E-06 | 2.80E-03 |
| 5198 | structural molecule activity | 4.07E-06 | 4.29E-03 |
| 30414 | peptidase inhibitor activity | 4.82E-06 | 5.08E-03 |
| 16798 | hydrolase activity, acting on glycosyl bonds | 5.26E-06 | 5.54E-03 |
| 46906 | tetrapyrrole binding | 5.27E-06 | 5.56E-03 |
| 61134 | peptidase regulator activity | 7.00E-06 | 7.38E-03 |
| 20037 | heme binding | 8.01E-06 | 8.45E-03 |
| 4866 | endopeptidase inhibitor activity | 8.79E-06 | 9.27E-03 |
| 9055 | electron carrier activity | 1.21E-05 | 1.28E-02 |
| 4364 | glutathione transferase activity | 2.36E-05 | 2.49E-02 |
| 15291 | secondary active transmembrane transporter activity | 2.42E-05 | 2.55E-02 |
| 15101 | organic cation transmembrane transporter activity | 2.59E-05 | 2.73E-02 |
| 5550 | pheromone binding | 2.89E-05 | 3.05E-02 |
| 4857 | enzyme inhibitor activity | 2.98E-05 | 3.14E-02 |
| 46873 | metal ion transmembrane transporter activity | 4.48E-05 | 4.72E-02 |
| 4568 | chitinase activity | 5.18E-05 | 5.46E-02 |
| 22804 | active transmembrane transporter activity | 8.21E-05 | 8.65E-02 |
| 4867 | serine-type endopeptidase inhibitor activity | 8.63E-05 | 9.10E-02 |
| 8194 | UDP-glycosyltransferase activity | 1.50E-04 | 1.58E-01 |
| 16765 | transferase activity, transferring alkyl or aryl (other than methyl) groups | 1.95E-04 | 2.06E-01 |
| 16490 | structural constituent of peritrophic membrane | 1.97E-04 | 2.08E-01 |
| 19842 | vitamin binding | 2.41E-04 | 2.54E-01 |
| 16758 | transferase activity, transferring hexosyl groups | 2.53E-04 | 2.67E-01 |
| 5549 | odorant binding | 4.34E-04 | 4.57E-01 |
|  |  |  |  |
| **GO ID** | **Biological Process** | **p** | **Bonferroni p** |
| 5975 | carbohydrate metabolic process | 3.69E-10 | 1.06E-06 |
| 6030 | chitin metabolic process | 7.32E-10 | 2.11E-06 |
| 6952 | defense response | 9.29E-10 | 2.67E-06 |
| 9607 | response to biotic stimulus | 1.04E-09 | 2.98E-06 |
| 6022 | aminoglycan metabolic process | 1.93E-09 | 5.55E-06 |
| 51707 | response to other organism | 2.21E-09 | 6.35E-06 |
| 6955 | immune response | 5.44E-09 | 1.57E-05 |
| 5976 | polysaccharide metabolic process | 7.55E-09 | 2.17E-05 |
| 6959 | humoral immune response | 1.87E-08 | 5.40E-05 |
| 6950 | response to stress | 2.90E-08 | 8.36E-05 |
| 9308 | amine metabolic process | 4.03E-08 | 1.16E-04 |
| 19730 | antimicrobial humoral response | 4.87E-08 | 1.40E-04 |
| 35080 | heat shock-mediated polytene chromosome puffing | 1.55E-07 | 4.48E-04 |
| 35079 | polytene chromosome puffing | 1.55E-07 | 4.48E-04 |
| 51704 | multi-organism process | 8.07E-07 | 2.32E-03 |
| 34605 | cellular response to heat | 1.07E-06 | 3.07E-03 |
| 6508 | proteolysis | 1.30E-06 | 3.75E-03 |
| 2376 | immune system process | 2.75E-06 | 7.91E-03 |
| 45087 | innate immune response | 3.32E-06 | 9.55E-03 |
| 19731 | antibacterial humoral response | 4.09E-06 | 1.18E-02 |
| 55114 | oxidation reduction | 4.19E-06 | 1.21E-02 |
| 16052 | carbohydrate catabolic process | 7.83E-06 | 2.25E-02 |
| 9617 | response to bacterium | 8.65E-06 | 2.49E-02 |
| 6635 | fatty acid beta-oxidation | 1.21E-05 | 3.47E-02 |
| 42742 | defense response to bacterium | 1.51E-05 | 4.36E-02 |
| 50802 | circadian sleep/wake cycle, sleep | 1.55E-05 | 4.46E-02 |
|  |  |  |  |
| **GO ID** | **Cellular Component** | **p** | **Bonferroni p** |
| 5576 | extracellular region | 3.64E-10 | 1.96E-07 |
| 5792 | microsome | 1.60E-06 | 8.63E-04 |
| 42598 | vesicular fraction | 1.60E-06 | 8.63E-04 |
| 5626 | insoluble fraction | 3.71E-06 | 2.00E-03 |
| 5624 | membrane fraction | 4.93E-06 | 2.65E-03 |
| 267 | cell fraction | 6.27E-06 | 3.37E-03 |
| 5615 | extracellular space | 1.39E-05 | 7.45E-03 |
| 42579 | microbody | 1.20E-04 | 6.45E-02 |
| 5777 | peroxisome | 1.20E-04 | 6.45E-02 |
| 43190 | ATP-binding cassette (ABC) transporter complex | 1.75E-04 | 9.40E-02 |
| 5956 | protein kinase CK2 complex | 4.13E-04 | 2.22E-01 |
