# Supplementary material for: The Functions of Grainy Head-Like Proteins in Animals and Fungi and the Evolution of Apical Extracellular Barriers
Source: PLoS One. 2012 May 9;7(5):e36254. doi: 10.1371/journal.pone.0036254 (PMC3348937; doi:10.1371/journal.pone.0036254)
Supplement: Text S1 — Statistical and Bioinformatical Analyses of Microarray Data. (DOC) [file pone.0036254.s007.doc]

**Text S1. Statistical and Bioinformatical Analyses of Microarray Data.**

Statistical analyses of the microarray experiments are composed of three steps: 1) normalization of microarray data, 2) sorting genes according to interest, and 3) statistical analyses of Gene Ontology terms over-represented in the sorted list of genes (for the *Drosophila* microarrays).

**1) Normalization of microarray data.**

The Agilent Feature Extraction Software (v10.5) provides high quality expression level reports for Agilent microarrays. Nevertheless, these data do need to be normalized in order to remove subtle biases due to variations in hybridization conditions or manufacturing. We normalized all samples simultaneously using the multiple-*loess* technique described elsewhere (Sasik et al. 2004).

**2) Sorting genes according to interest.**

In designing the interest statistic we borrowed ideas from Cole et al. (2003) and their software package Focus. The interest statistic reflects the understanding that a gene with a greater fold change than another gene (in terms of absolute value) is likely to be more interesting and informative. Also, given two genes with the same fold-change values, the gene with the higher expression level (and therefore a higher absolute change) is likely the more interesting one.

**3) Statistical analyses of Gene Ontology terms.**

We note that the sorted gene list created in Step 2 does not imply statistical significance for any individual gene; however, it does contain information on which *groups of genes* or pathways might be responsible for the phenotypes of interest. Due to the way the genes are sorted, it is reasonable to expect that genes responsible for the phenotype might be ranked higher (on average) than genes unrelated to the phenotype. To quantify the statistical significance of a group of genes, we first transform the integer ranks into real ranks as follows:
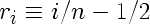
, where *i* is the integer rank of a gene, and *n* is the total number of genes on the list (the first gene on the list has integer rank *n* and a real rank ½). For large values of *n*, ranks
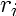
 are quasi-continuous real numbers uniformly distributed between -½ and ½. Now let us suppose that there is a group of *m* functionally related genes (these genes may be part of the same pathway or Gene Ontology term, or simply part of a group we wish to test for significance). Let their real ranks be
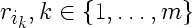
, and let us define a rank-sum statistic:


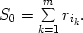


The significance of this group of genes is given by the probability that a score greater or equal to
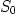
 will be realized for *m* genes that are truly unrelated to the phenotype. To this end, we assume a null model in which the real ranks of the *m* genes are iid (independently and identically distributed) random variables drawn from a uniform distribution of the interval -½ to ½. This null model is entirely realistic when the gene rankings are determined solely by measurement errors. For example, comparing two identical RNA samples and sorting genes by the apparent differences (which are false) falls into this category. For large *m* we use the central limit theorem of statistics in the form:


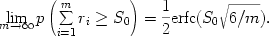


We use the formula on the right-hand side to estimate the *p-*value of a group of genes whenever *m* ≥ 3, and use the exact formulas for *m* = 1 and 2. Since a typical pathway involves more than three genes, this asymptotic formula is very accurate; even for cases where *m* = 3, the asymptotic formula works very well. This group analysis is very powerful, because a group may be statistically significant even when none of its members is particularly highly ranked. This is due to the exponential asymptotic dependence of *p* on *m*:


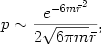


where
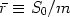
 is the mean rank of the group. It is now easy to see that a large group of genes may be significant even if none of its genes appear among, say, the top 100 on the sorted list. The *p*-value defined here has been tested on numerous artificial data sets in which groups of genes were mixed with background genes at various dilutions, and it has performed as desired.

We calculate *p*-values for groups of genes by performing Gene Ontology (GO) analysis (www.geneontology.org). All GO terms define groups of genes for which we calculate *p*-values. Because of the hierarchical structure of the GO database (it is a directed acyclic graph in which the nodes are GO terms), every term contains all the genes of its daughter terms and the daughter terms are not independent of the parent term(s). It is therefore possible, and indeed likely, that a term may appear statistically significant because its parent or daughter term is statistically significant. It is not clear which term, or both, should be reported. The parent-daughter dependence poses a problem for a purist’s *p*-value interpretation, but in practice it does not matter whether the same biological process gets reported multiple times. A heuristic method which eliminates the problem of parent-daughter dependence on the GO graph has been proposed by Ogawa et al (2004). The parent-daughter dependence means the same biological process may get reported multiple times which makes it difficult to meaningfully adjust the *p*-values for multiple testing. We find that a simple Bonferroni adjustment is somewhat useful, even though it is very conservative.

**REFERENCES**

Cole SW, Galic Z, Zack JA (2003) Controlling false-negative errors in microarray differential expression analysis: a PRIM approach. Bioinformatics 19(14): 1808–1816.

Ogawa S, Lozach J, Jepsen K, Sawka-Verhelle D, Perissi V, Sasik R, et al. (2004) A nuclear receptor corepressor transcriptional checkpoint controlling activator protein 1-dependent gene networks required for macrophage activation. Proc Natl Acad Sci USA 101(40): 14461–14466.

Sásik R, Woelk CH, Corbeil J (2004) Microarray truths and consequences. J Mol Endocrinol 33: 1–9.

Selitrennikoff CP, Nelson RE, Siegel RW (1974) Phase-specific genes for macroconidiation in *Neurospora crassa*. Genetics 78(2): 679–690.
